# Catalyst screening for electrochemical ammonia synthesis: a critical review

**DOI:** 10.1039/d5na01170a

**Published:** 2026-04-27

**Authors:** Dominik G. Jammal, Ricardo Bernardino, Nuno Canha, Cristina M. Cordas, Rui P. P. L. Ribeiro

**Affiliations:** a HyLab- Green Hydrogen Collaborative Laboratory, Sines Central Termoelétrica Estrada Nacional 120-1 Sines 7520-089 Portugal dominik.jammal@hylab.pt; b LAQV-REQUIMTE, Department of Chemistry, NOVA School of Science and Technology, NOVA University Lisbon Caparica 2829-516 Portugal

## Abstract

Ammonia (NH_3_) is a promising carbon-free energy carrier, and its synthesis is a key process in the chemical industry. While the Haber–Bosch process remains dominant, alternative approaches such as lithium-mediated nitrogen reduction (Li-mNRR), electrocatalysis, and photocatalysis are increasingly explored for sustainable NH_3_ production. In this review, we systematically analyze 215 catalytic systems, evaluating production rates, faradaic efficiencies, and cost-performance. Key trends highlight the importance of transition-metal centers (*e.g.*, Mo, Ni, Cu), high-surface-area conductive supports (MOF- or MXene-based), and structural optimization *via* porosity, defect engineering, and doping. Our analysis identifies major gaps in standardized data reporting, particularly the frequent omission of turnover frequency, stability, and surface area, which hinder meaningful comparisons and limit machine-learning-driven catalyst design. We propose standardized metrics (*e.g.*, µmol cm^−2^ s^−1^) and comprehensive reporting of key parameters to enable cross-catalyst comparison and the development of high-quality datasets. These insights provide practical guidelines for the rational design of efficient, stable, and scalable catalysts, with Mo-based systems, MOFs, and transition-metal nitrides/carbides emerging as particularly promising candidates for electrochemical and photocatalytic NH_3_ synthesis.

## Abstract 2

Mo-based catalysts, MOFs, and TM-nitrides and carbides are identified as leading NRR systems, with integrated qualitative and ML-analyses elucidating design rules that govern activity, stability, and cost-efficient electrochemical NH_3_ synthesis.

## Graphical abstract

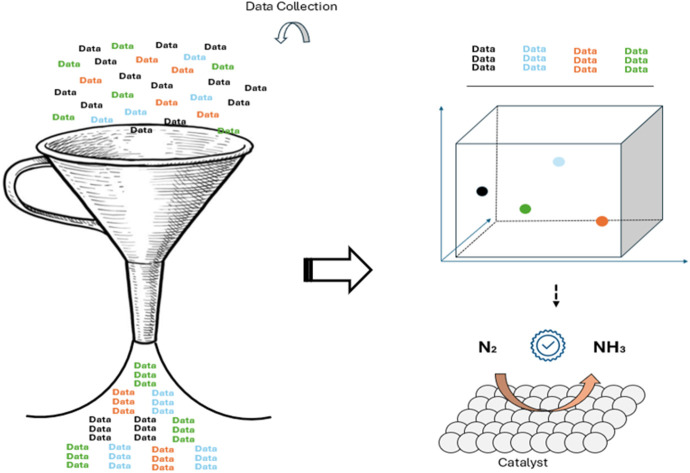

## 1 Introduction

**Fig. 1:**
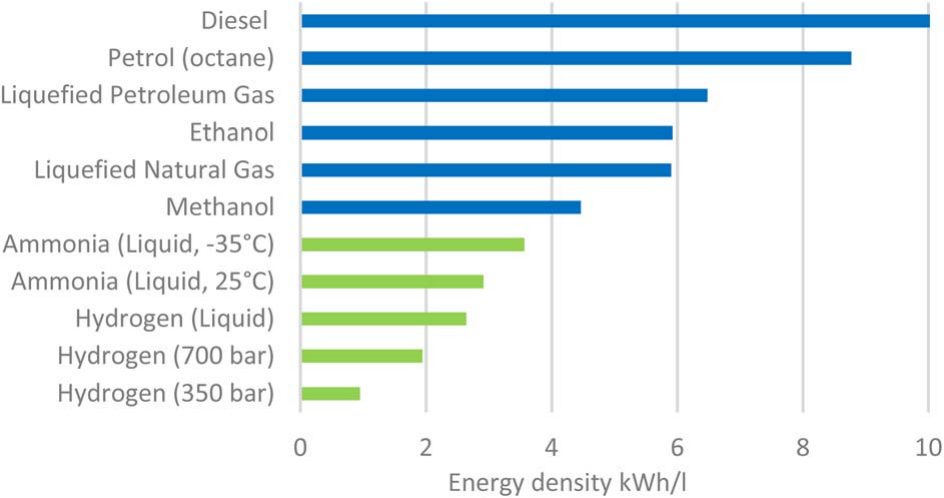
Comparison of energy densities of zero carbon fuels (green) and carbon-based fuels.^11^ Created using data reported in ref. 11. The Royal Society (2020).

Ammonia (NH_3_) is one of the most crucial chemicals in the global economy of the 21st century.^1^ It is primarily used as a precursor for fertilizers and other essential industrial chemicals.^2^ The importance of NH_3_ is further underscored by its key role in sustaining global food production, with 40–50% of the global food supply depending on NH_3_-based fertilizers.^3,4^ With the population of the world projected to grow by approximately 9.8 billion people by 2050,^5^ ammonia's significance in ensuring future food security will continue to increase.^6^ Beyond its agricultural and industrial uses, ammonia is increasingly recognized as a promising hydrogen carrier and carbon-free energy vector.^7^ With a hydrogen content of 17.6% by weight and existing infrastructure for storage and transport, NH_3_ offers distinct advantages over molecular hydrogen (H_2_), which requires either cryogenic (−253 °C) or high-pressure (up to 700 bar) storage. Its ease of liquefaction (−33.4 °C, 8–10 bar) and well-established distribution networks make NH_3_ particularly attractive for large-scale energy applications.^8,9^ Ammonia can also be directly utilized without prior cracking back to hydrogen, for example in microturbines or in ammonia fuel cells, where ongoing research demonstrates its potential as a zero carbon fuel.^10^ While the exact comparison is case-dependent, some studies report that the energy penalty of converting H_2_ to NH_3_ and back is of the same order of magnitude as liquefying H_2_. In addition to its storage and handling advantages, ammonia exhibits a favorable energy density compared to other carriers, and in particular H_2_. In fact, liquefied hydrogen (LH_2_) has an energy density of 2.36 kWh L^−1^, whereas liquefied ammonia (LNH_3_) reaches 3.53 kWh L^−1^, representing a 33% higher volumetric energy content. Compared to carbon-based carriers like methanol (LMeOH = 4.39 kWh L^−1^), ammonia provides a zero-carbon alternative with well-established logistics and reduced CO_2_ emissions as depicted in Fig. 1.^11^ These characteristics further highlight the potential of NH_3_ as a versatile and sustainable candidate for both large-scale energy storage and direct energy use in a future hydrogen economy.^12^ Ammonia production is commonly categorized into three main routes: grey, blue, and green ammonia. Grey ammonia is obtained *via* conventional steam methane reforming (SMR) combined with the Haber–Bosch process, but it is associated with substantial CO_2_ emissions. Blue ammonia follows the same production pathway yet integrates carbon capture and storage (CCS) technologies, lowering CO_2_ emissions by approximately 9–12%.^13^ In contrast, green ammonia represents a paradigm shift, as it relies on renewable energy sources such as wind, solar, or hydropower for hydrogen generation through electrolysis, and subsequent reaction with nitrogen (N_2_), thereby offering a near carbon-free alternative.

**Fig. 2:**
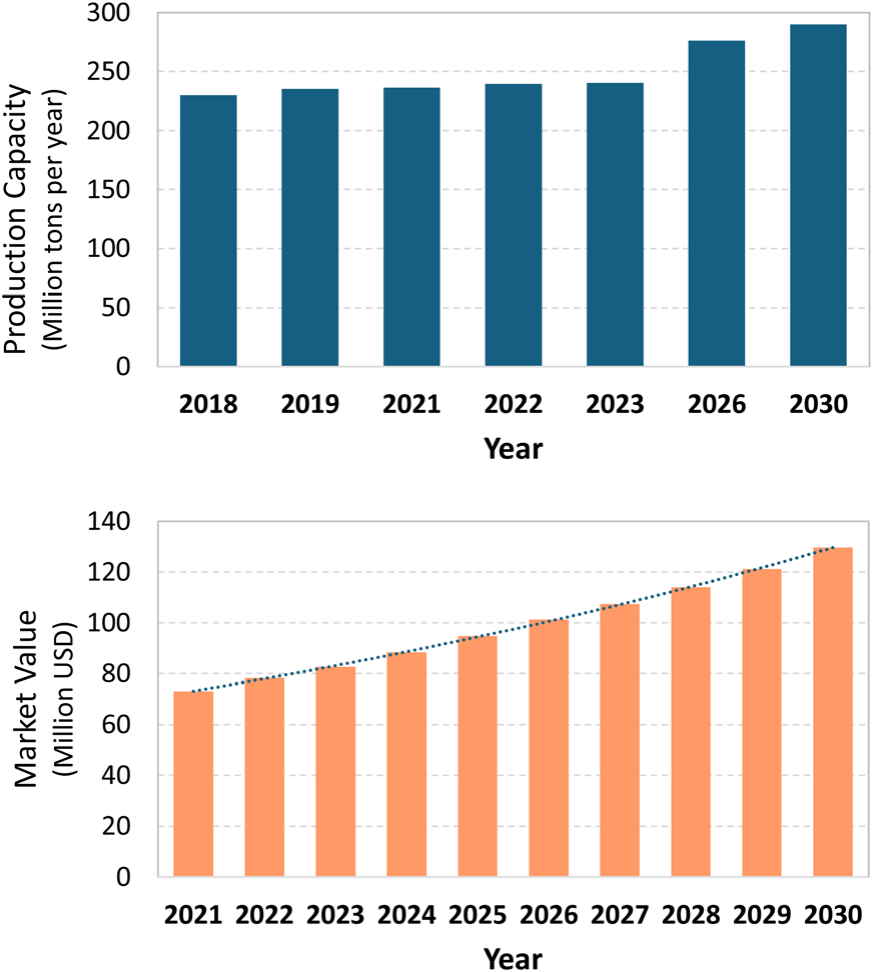
NH_3_ Production capacity in million tons per year from 2018 to 2023 (above), along with forecasts for 2026 and 2030 (above). Ammonia market value in billions USD from 2021 to 2024, Best wishes, Joe Busby along with projection to 2030 (down).^15^ Created using data reported in ref. 15. IEA (2021). Licence: CC BY 4.0.

To contextualize these advantages, it is useful to consider the current scale of ammonia production and the industrial processes that enable it. As depicted in Fig. 2, the global NH_3_ production in 2023 reached *c.a.* 240 million tons annually, with a market value of USD 83.36 billion. This is expected to grow to around 290 million tons by 2030, reaching an estimated value of USD 129.63 billion.^14,15^

As mentioned, ammonia is industrially produced through the Haber–Bosch process since the 1910's, although throughout the 20th century, the process underwent continuous improvements, including a transition from coal to natural gas as the source for H_2_ production.^16^ Despite ongoing research into more eco-friendly methods, such as hydrogen production through electrolysis, the Haber–Bosch process using natural gas remains the industry standard due to its low costs and an overall energy efficiency of 66%.^17^ The Haber–Bosch process involves reacting nitrogen obtained from air, typically through cryogenic air separation (ASU) or pressure swing adsorption (PSA), with hydrogen derived from natural gas, being described through the following exothermic reaction.^18^

1 N_2_(g) + 3H_2_(g) ⇌ 2NH_3_(g) Δ*H*^0^ = −92.6 kJ mol^−1^

**Fig. 3:**
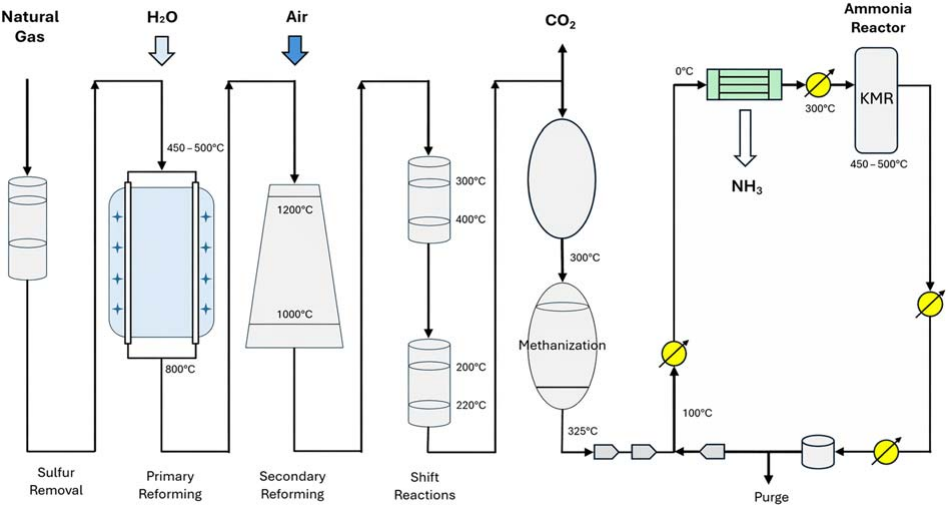
Process flow diagram of the SMR reforming based Haber–Bosch process: Key stages from natural gas desulfurization, primary and secondary reforming and shift reactions to the methanation and ammonia synthesis loop.^25,26^ Adapted from ref. 26 with permission Elsevier

The formation of NH_3_ is thermodynamically favoured at low temperatures. However, kinetic limitations under those mild conditions, implies the industrial Haber–Bosch process to be carried out at temperatures between 400–500 °C to achieve an efficient production rate.^19,20^ The extremely high activation energy (*E*_a_) required to break the triple bond (945 kJ mol^−1^) necessitates these high temperatures to facilitate efficient dissociation.^21^ Equilibrium calculations reveal that at temperatures above 400 °C and a pressure of 0.1 MPa, more than 99% of NH_3_ decomposes.^22^ To address this challenge, the Haber–Bosch process operates under a compromise, utilizing moderately high temperatures of 400–500 °C combined with high pressures of 20–40 MPa.^23^ High operating pressures shift the equilibrium towards NH_3_ production, while the temperatures are sufficient to overcome the kinetic barrier without causing excessive decomposition. Typically, only about 15% of the reactants convert to NH_3_ in a single reaction pass, but the unreacted N_2_ and H_2_ are recycled back into the Haber–Bosch production cycle. This recycling approach, combined with the integration of heat recovery systems, leads to an overall conversion rate of ∼97%. The produced NH_3_ is liquefied at −33.4 °C and maintained under pressures of 8–10 bar to ensure safe storage and distribution.^24^Fig. 3 shows the process flow diagram of the Haber–Bosch process based on steam methane reforming.^25,26^

The high efficiency of this process is due to both optimal reaction conditions but also the utilization of highly effective catalysts that significantly enhance NH_3_ production. In particular, iron (Fe) and nickel (Ni) catalysts are widely used in the process because of their catalytic activity, availability, and cost-effectiveness (Fe ∼ $107 per ton, Ni ∼ $16,300 per ton).^18,26–28^ The incorporation of ruthenium (Ru) catalysts also proves to be beneficial in the Haber–Bosch process as these catalysts are highly efficient owing to their exceptional selectivity and long-term stability.^26,29^ Their main drawback is their high cost (*e.g.* Ru ∼ $13 million per ton). Although a range of d-metal catalysts—including Co, Cr, Mn, Mo, Pd, Pt, Rh, W, and V—are under continuous investigation, Fe and Ni are particularly notable for their favourable balance between catalytic performance and cost, whereas Ru is distinguished mainly by its superior intrinsic activity. Typically, these catalysts are combined with various activators and support materials to enhance their performance. Alumina (Al_2_O_3_) and potassium oxide (K_2_O) for instance serve as activators to improve the catalysts' efficiency and stability.^27^ Additionally, support materials like alumina (Al_2_O_3_) and silicon dioxide (SiO_2_) increase the catalyst's surface area, thereby enhancing overall effectiveness by improving reactant accessibility and promoting better dispersion of the active sites.^30–32^

### 1.1 Efficiency and CO_2_ emissions of the Haber–Bosch process

**Fig. 4:**
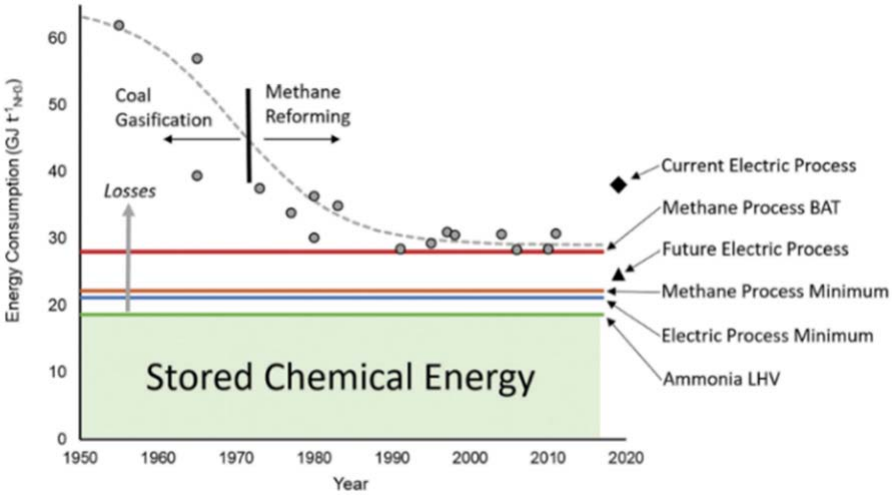
Efficiency improvements in NH_3_ production are shown, comparing real plant data with BAT, energy minima for methane- and electrolysis-based routes (H₂, LHV basis), and current versus projected electric processes. LHV indicates the lower heating value of NH_3_, with excess energy reflecting losses.^33^ Reproduced from Ref. 33 via Creative Commons CC BY 3.0 licence.

The Haber–Bosch process, utilizing H_2_ from natural gas through Steam Methane Reforming (SMR), remains the more economically viable option. Continuous improvements in this process have achieved an energy consumption of 28 GJ per ton of NH_3_ (with a practical minimum around 22 GJ per t NH_3_ (Fig. 4)).^33^ With global natural gas prices ranging from $0.00728 to $0.0425 per kWh, the cost of producing one ton of NH_3_ is estimated to be between approximately $44 and $330.^34,35^ However, the high CO_2_ emissions associated with this process—approximately 2.4 tons per ton of NH_3_, accounting for about 1% of global CO_2_ emissions—underscore the need for cleaner ammonia production methods.^36^ With green H_2_ produced by water electrolysis, CO_2_ emissions can be dramatically reduced. While the current electrolysis-based Haber–Bosch process consumes around 38 GJ per t NH_3_ in practice, the theoretical minimum energy required for NH_3_ synthesis itself is approximately 21 GJ per t NH_3_. This theoretical value represents the energy needed solely for the chemical conversion of N_2_ and H_2_ into NH_3_ and is lower than the practical minimum of the conventional HB process because it does not include energy losses from H_2_ production.^36^

As renewable energy prices continue to decline—currently around USD $0.0490 per kWh for photovoltaic (PV) power—the estimated cost of producing green NH_3_ is projected to be approximately USD $520 per t NH_3_.^35^ Despite these advancements, independently from the H_2_ source, the Haber–Bosch process remains energy-intensive due to the high temperature and pressure requirements. Furthermore, the optimal Haber–Bosch process operates continuously which adds complexity when working with intermittency of the conversion of renewables into H_2_.^37^ Consequently, there is an ongoing effort to develop alternatives to the HB process. A promising approach is the production of NH_3_ through (photo)-electrocatalysis, as these methods offer a cleaner alternative with potential reduction of CO_2_ emissions from 1.6 tons to around 0.1 tons per ton of NH_3_. A comparative overview of the performance of conventional and emerging NH_3_-production routes is provided in Table 1, which summarizes the most relevant efficiency metrics and operational characteristics. These alternative approaches will be further explored in Chapter 3.^38^

**Table 1:** Comprehensive evaluation of the process performance of different ammonia production technique

Process performance overview	SMR HB-process	Benchmark electrolysis HB-process	Electrochemical synthesis (aqueous NRR purge)	Lithium mediated NRR (Li-mNRR)
Energy consumption per ton of NH_3_^a^ (GJ per kWh)	28/7778 (ref. 39)	38.4/11 000 (ref. 39)	∼67/19 000 (ref. 39)	190/50 000 (ref. 39)
Efficiencies (%) b	61–66 (ref. 40)	65	Not reported	Not reported (ref. 41)
**Faradaic** efficiencies (%)	∼97 (ref. 42)	Not reported	**∼73** (ref. 42)	**78.2** d ^,^^43^
Costs (USD $ per t NH_3_) c	55–330	520	910	2500
CO_2_ emissions per t NH_3_	2.4	0.1	0.1	0.1
TRL	9	7–9	1–3	1–3

a Energy consumption values include all major process operations: heating, distillation, the NRR electrolyzer, the H_2_ electrolyzer, air separation units, and O_2_ compression.

b Overall efficiencies for electrochemical ammonia synthesis and lithium-mediated NRR are not provided, as standardized and reproducible efficiency metrics for these emerging technologies are not yet available.

c Reported costs reflect the energy consumption in kWh per ton of NH_3_, calculated using the current electricity price of USD 0.0490 per kWh as well as Natural gas price range (0.0073–0.0425 USD $ per kWh).

d Ambient conditions.

### 1.2 State-of-the-art and scope of this review

In recent years, several comprehensive review articles have addressed electrochemical and photo(electro)chemical nitrogen reduction reactions (NRR), reflecting the rapidly growing interest in sustainable ammonia synthesis. Key reviews published between 2022 and 2025 have focused on distinct but complementary aspects of this research. For instance, Ahmed *et al.*, 2023 (ref. 44) and Mangini *et al.* (2024)^45^ systematically analyzed Li-mNRR, discussing catalysts, electrolyte formulations, reactor designs, and strategies to enhance faradaic efficiency. Biswas *et al.* (2022)^46^ and Mahmood *et al.* (2024)^47^ focused on catalyst design principles for electrochemical NRR, mechanistic insights, and performance benchmarking using transition metal-based systems. Meanwhile, Ješić *et al.* (2024)^48^ and Shen *et al.* (2022)^49^ examined photo(electro)catalytic routes, highlighting the role of light absorption, charge carrier dynamics, and defect engineering in enhancing catalytic activity. In addition, emerging material classes such as metal–organic frameworks (MOFs) have gained increasing attention due to their high surface areas, structural tunability, and the large density of accessible catalytic sites, which are particularly beneficial for N_2_ adsorption and activation. The growing relevance of MOFs for electrocatalytic NRR has been comprehensively summarized in “*Recent Advances in Metal–Organic Frameworks and Their Derivatives for Electrocatalytic Nitrogen Reduction to Ammonia*” (2022), highlighting both pristine MOFs and MOF-derived materials as promising catalyst platforms. In the present review, the analysis is intentionally limited to pristine MOF-based catalysts. MOF-derived materials with substantially altered compositions or morphologies are excluded, as their complex transformation pathways and heterogeneous structures hinder a consistent and quantitative comparison of production rates (PR), faradaic efficiencies (FE), and catalyst costs (CC) across different NH_3_ synthesis routes.

While these prior reviews provide valuable insights into catalyst development, reaction mechanisms, and reactor concepts, they predominantly focus on either catalytic activity metrics or mechanistic trends, with techno-economic aspects largely treated qualitatively. In contrast, the present work systematically integrates PR, FE, and CC for 215 catalytic systems, enabling direct, cross-platform comparison across lithium-mediated, electrochemical, and photo(electro)catalytic NRR systems. To our knowledge, no previous review has benchmarked such a large number of catalytic systems across multiple quantitative performance metrics, including techno-economic considerations. By adopting a harmonized, quantitative assessment, this review provides a coherent overview of current progress, elucidates performance trade-offs, identifies promising catalyst-process combinations, and establishes a structured basis for guiding future research toward scalable, energy-efficient, and economically viable NH_3_ production technologies.

### 1.3 Selection criteria and methodology for catalytic systems

The 215 catalytic systems analyzed in this review were selected based on a systematic, literature-driven approach. The initial basis was established from recent review articles, particularly the work of Castillejos and Garcia-Bordejé (2024),^50^ which provided an overview of relevant catalysts and reaction routes. Building upon this foundation, primary research articles published between 2022 and 2025 were screened to capture the most current developments in Li-mNRR, electrochemical, and photo(electro)catalytic NRR. Inclusion criteria required that each system have verifiable literature references consistent with the source review tables, clearly reported performance metrics (PR, FE, and, where available, CC), and applicability to contemporary research directions. Systems were excluded if literature references were inconsistent, data were incomplete, or the study predated 2022, ensuring a focus on the state-of-the-art. This methodology enabled the compilation of a large, comparable dataset, allowing the identification of performance trends, trade-offs, and promising catalyst-material combinations across different NRR approaches. The SI (Table S1) provides a comprehensive overview of all 215 collected catalysts, including the original literature references and the normalized performance scores derived from PR, FE and CC.

## 2. Fundamentals of ammonia synthesis

The development of improved approaches for ammonia synthesis must be supported by a strong understanding of the process fundamentals. Advances in catalysts and novel optimized synthetic methods are built upon the underlying mechanistic steps, key inspirations, and thermodynamics of NH_3_ synthesis.

### 2.1 Nature-inspired enzymes

**Fig. 5:**
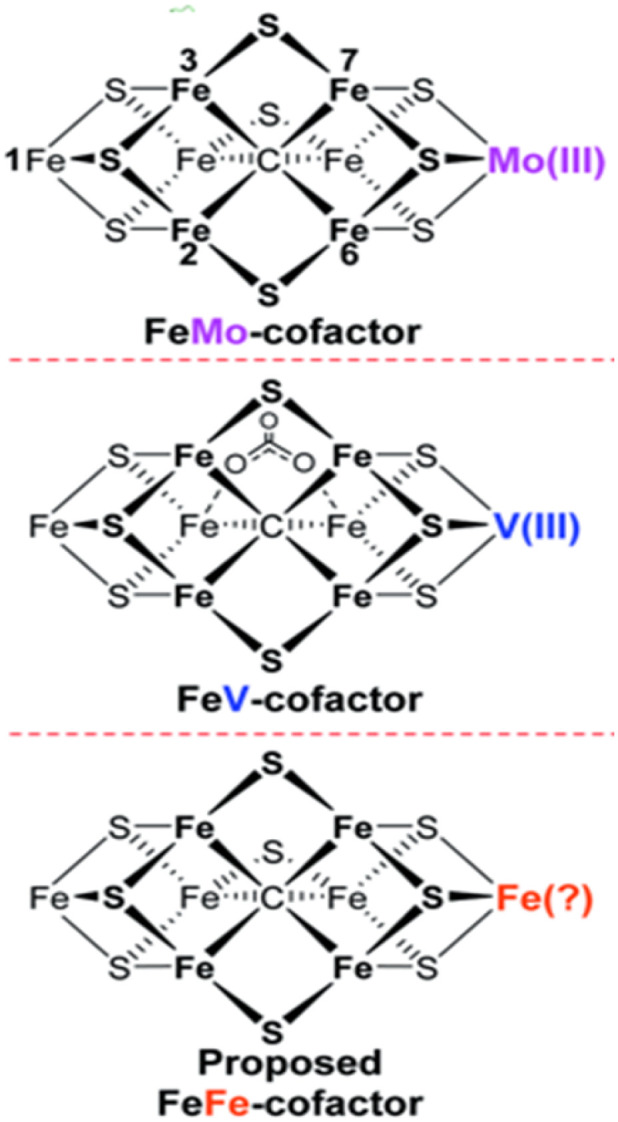
Fe-Mo-S nitrogenase, Fe-V-S-nitrogenase and Fe-Fe-S-nitrogenase.^54^ Adapted from ref. 54*via* Creative Commons CC BY 3.0 licence.

Nitrogen fixation is a natural process in which gaseous N_2_ undergoes chemical reactions to be transformed into valuable compounds such as ammonia, nitrates, or nitrites. In biological systems, one of the enzymes catalysing such processes is nitrogenase that catalyzes H_2_ reduction into NH_3_ with sunlight serving as the driving force for the reactions shown in eqn (2.1)–(2.3).^51,52^ These enzymatic nitrogen reduction reactions (NRR) demonstrate remarkable efficiencies up to 66%, utilizing, in the case of the Fe–Mo nitrogenase (nitrogenase using an iron–molybdenum (FeMo cofactor) structure as the active site), eight protons, eight electrons and 16 equivalents of Adenosine triphosphate (ATP) to convert one mol of N_2_ into two moles of NH_3_.^53^ The energy requirement for this conversion is 244 kJ mol^−1^, which is significantly lower than the 520 kJ mol^−1^ needed for the Haber–Bosch process, highlighting the superior energy efficiency of biological nitrogen fixation. Iron (Fe), molybdenum (Mo), and vanadium (V) are recognized as crucial metal centers in biological nitrogen fixation. Fig. 5 shows the three main types of nitrogenase enzymes' active centers: Fe–Mo–S, Fe–V–S, and Fe–Fe–S nitrogenases.^54^ Although their cluster structures are identical, they differ in the metals in the catalytic active sites, leading to varying energy requirements for the N_2_ fixation.^50,53^

Due to the highly selective and efficient conversion of N_2_ to NH_3_ by these enzymes, there is increasing interest in mimicking nitrogenase enzymes for synthetic NH_3_ production.^51,52^ Research is now focused on replicating these enzymes with either heterogeneous or homogeneous (electro-)catalysts to understand and utilize their catalytic sites, structures and mechanisms, aiming to achieve similar high selectivity and efficiency.^55^ These enzymatic insights are not only relevant from a biological perspective but also serve as guiding principles for synthetic catalyst design. To translate this understanding into practical applications, theoretical approaches such as Density Functional Theory (DFT) have been applied to elucidate the fundamental reaction mechanisms of NRR.^56^

2.1 Mo-nitrogenase: N_2_ + 8H^+^ + 8e^−^ + 16ATP → 2NH_3_ + H_2_ + 16ADP + 16P

2.2 V-nitrogenase: N_2_ + 12H^+^ + 12e^−^ + 24ATP → 2NH_3_ + 3H_2_ + 24ADP + 24Pi

2.3 Fe-nitrogenase: N_2_ + 24H^+^ + 24e^−^ + 48ATP → 2NH_3_ + 9H_2_ + 48ADP + 48Pi

#### 2.1.1 Mechanism of the NRR

**Fig. 6:**
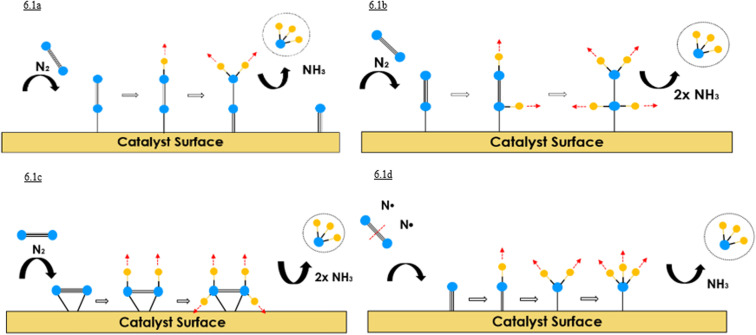
General NRR pathways highlighting coordination modes and binding interactions.^50,53^

A detailed understanding of the reaction pathways in NRR is essential to enable its optimization. Therefore, DFT calculations have been employed, allowing for the identification of key transition states and the rate-determining step.^57^ Calculations of the free energy differences (Δ*G*) for the elementary reactions provided valuable insights into the stability of the system and possible surface intermediates.^58^ These theoretical results were consistent with experimental observations and led to the classification of two general reaction pathways, based on coordination sites and specific binding interactions at the catalytic surface: (I) the associative pathway, which can be further divided into distal (Fig. 6a), alternating (Fig. 6b), and enzymatic (Fig. 6c) mechanisms, and (II) the dissociative pathway (Fig. 6d).^59^ The coordination mode of N_2_—end-on or side-on—plays a crucial role in molecule–surface interactions and the design of efficient metal catalysts. Distal and alternating pathways are typically associated with side-on adsorption, whereas the enzymatic pathway proceeds *via* an end-on configuration. Insights from DFT calculations and adsorption studies have elucidated the corresponding free-energy profiles, although the detailed mechanism of nitrogen fixation remains not fully understood.^58–61^

##### 2.1.1.1 Associative pathway

In the associative pathway, N_2_ adsorbs onto the catalytic surface and undergoes stepwise protonation and hydrogenation, facilitated by the catalyst's provision of electrons and protons.

This pathway comprises three distinct mechanisms: distal, alternating, and enzymatic.^58^ In the distal pathway (Fig. 6a), N_2_ is adsorbed in a side-on configuration and sequentially hydrogenated. Initial protonation elongates the N–H bond, weakening the N≡N triple bond and forming intermediates such as diazenido, nitrido, and imido species. The first NH_3_ molecule desorbs, followed by a second cycle of protonation and NH_3_ formation.^59–61^ The alternating pathway (Fig. 6b) involves hydrogenation of both nitrogen atoms in an alternating manner. The process involves both side-on and end-on interactions of H_2_, leading to symmetric and asymmetric weakening of the N≡N bond. Key intermediates include diazene, hydrazido, and hydrazine, with N_2_H_4_ formation identified as a potential rate-limiting step.^58,59^ The enzymatic pathway (Fig. 6c) closely resembles the alternating mechanism but proceeds *via* end-on coordination of N_2_ to the nitrogenase active site. Electron and proton transfer is facilitated by the enzyme, gradually converting N_2_ into NH_3_. The reduction of 
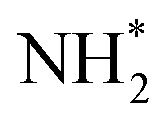
 to NH_3_ is suggested as the rate-limiting step.^58^

##### 2.1.1.2 Dissociative pathway

In the dissociative pathway, the N≡N bond is completely cleaved upon adsorption, requiring significant energy (∼945 kJ mol^−1^). Subsequent hydrogenation of individual nitrogen atoms leads to NH_3_ formation. Although feasible, this pathway is energetically less favourable compared to associative mechanisms.^58,60^

### 2.2 N_2_-binding

**Fig. 7:**
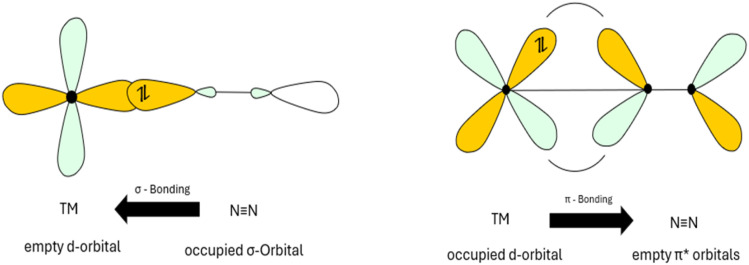
Metal–ligand bonding interactions between transition metals and nitrogen.

Understanding how N_2_ interacts with metal catalysts—including different binding modes and their energy requirements—enables the targeted selection and optimization of catalysts for specific applications, enhancing activity and clarifying reaction mechanisms. This optimization strategy requires a systematic analysis of catalytic materials, which can be classified into four categories: (a) non-noble metals, (b) noble metals, (c) alkali metals and (d) metal–organic compounds. The aim is to gain insight into the interaction between catalysts and N_2_, with a focus on optimizing adsorption and desorption characteristics. For efficient NRR, catalysts need to have well-balanced binding properties—neither too weak nor too strong—to ensure optimal performance. The use of (a) non-noble d-metals as catalysts is inspired by natural processes (*e.g.*, Fe, Mo, V) and has proven effective in industrial applications such as the Haber–Bosch process (*e.g.*, Fe).^52^ Their catalytic activity originates from the destabilization of the N≡N bond through effective orbital overlap between the metal and the nitrogen atom, which enhances electron density at the metal's d-orbitals and facilitates bond weakening.^62^ Among the d-metals explored in NRR, titanium (Ti), vanadium (V), chromium (Cr), iron (Fe), cobalt (Co), nickel (Ni), and molybdenum (Mo) are particularly valuable due to their electronic properties, which are crucial for N_2_ activation and subsequent reduction.^63^ The interaction mechanism of these metals follows the principles of metal–ligand bonding.^64^ Specifically, σ-donation from the sp-hybridized orbitals of nitrogen contributes with electron density to the vacant d-orbitals of the metal centre, while π-back bonding occurs through interactions between the metal's filled d-orbitals and the nitrogen's empty π* orbitals. This synergistic combination of σ-donation and π-back bonding reduces the electron density of the N_2_ triple bond, leading to its elongation and weakening. Consequently, the molecule becomes more susceptible to activation and reduction (Fig. 7).^64,65^

Among these categories, (b) noble metals such as ruthenium (Ru), rhodium (Rh), palladium (Pd), iridium (Ir), osmium (Os), platinum (Pt), gold (Au), and silver (Ag) follow the same metal–ligand bonding principles as non-noble transition metals. Although their orbital overlap resembles that of non-noble metals, their unique electronic structures give rise to distinct bonding characteristics. Owing to their filled d-orbitals, noble metals display reduced reactivity toward air and moisture, which enhances their electronic stability and lowers their tendency to undergo undesired side reactions. These properties make noble metals valuable in catalytic applications, including the Haber–Bosch process, where ruthenium-based catalysts have been widely applied for improved performance.^66^ In contrast, (c) alkali metals such as lithium (Li), sodium (Na), and potassium (K) represent a different type of catalyst. Their interaction with nitrogen is not governed by classical covalent orbital overlap, but rather by ionic interactions. Among them, lithium stands out due to its high reactivity and efficiency in NRR, whereas sodium and potassium, although catalytically active, generally exhibit lower performance. The oxidation of lithium into Li^+^ ions facilitate the electron transfer to N_2_, weakening the N≡N triple bond and enabling the formation of Li–N complexes and subsequent Li_*x*_N_*y*_H_*z*_ intermediates. These intermediates play a crucial role in further transformation steps, ultimately leading to the formation of NH_3_.^67,68^ In contrast to both transition and alkali metals, (d) hybrid metal–organic compounds (MOCs) represent a fundamentally different class of catalysts, in which the metal centre is coordinated by organic ligands that strongly influence its electronic properties and catalytic activity. MOCs encompass a wide range of molecular structures, with Metal–Organic Frameworks (MOFs) and metal-porphyrins being prominent examples due to their stability and tunable properties.^69,70^ The activation of N_2_ in metal–organic compounds involves several key steps in the binding mechanism. Initially, the metal centre binds the N_2_ molecule through coordinative interactions, typically in either the η^1^ (end-on) or η^2^ (side-on) mode, depending on the electronic and steric properties of the complex. In the η^1^ mode, N_2_ binds *via* one nitrogen atom, whereas in the η^2^ mode, it coordinates through both nitrogen atoms. These binding modes enable electron donation from the metal to the N_2_ molecule, weakening the strong N≡N triple bond and altering its electronic structure. This activation enhances the reactivity of N_2_, facilitating subsequent chemical transformations. In catalytic systems for ammonia synthesis, further steps such as stepwise protonation led to NH_3_ formation. The ability of metal–organic catalysts to weaken the N≡N bond and promote protonation is critical in emerging electrocatalytic and photocatalytic approaches for sustainable ammonia production.^71,72^

## 3 Emerging green ammonia production technologies

Modern ammonia synthesis aims to reduce CO_2_ emissions and energy consumption compared to the traditional Haber–Bosch process. The following sections present four of the most promising methods for greener ammonia production.

### 3.1 Mechanochemistry

**Fig. 8:**
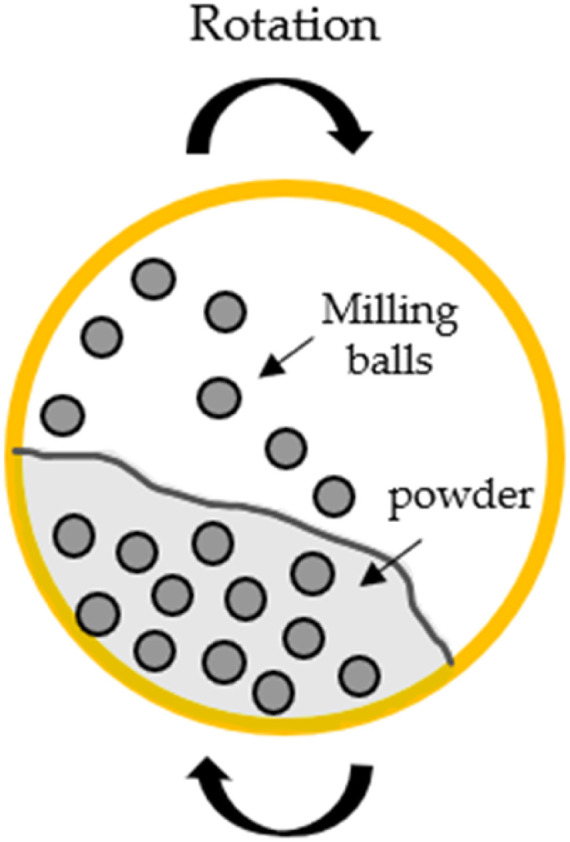
Schematic representation of ball milling for NH_3_ synthesis.^73^ Adapted from ref. 73*via* Creative Commons CC BY 4.0 licence. © 2022 by the authors. Licensee MDPI, Basel, Switzerland.

Mechanochemistry enables chemical reactions by applying mechanical force, commonly through processes like ball milling. In this approach, a rotating ball mill activates a solid catalyst (commonly Fe, Co, or Ru), often promoted with alkali metals such as Na, Cs, or K. The reactants, either solid, liquid, or gaseous, are introduced into the milling vessel, where reactions occur on the surface of the activated catalyst. Fig. 8 illustrates the working principle, showing how mechanical activation facilitates these reactions.^73^ Interest in mechanochemistry for ammonia synthesis is based on its advantages over traditional methods, operating under milder conditions with significantly lower temperature and pressure than the Haber–Bosch process. Additionally, this method avoids the use of solvents, making it more environmentally sustainable and scalable. In 2021, J-B. Baek *et al.*^74^ reported significant advances in mechanochemical ammonia synthesis using ball milling with an Fe-based catalyst. The mechanical impacts generate a high density of surface defects, which markedly improves the activation and dissociation of N_2_. During the subsequent hydrogenation steps, these activated sites promote the formation and release of NH_3_. Under mild conditions (45 °C and 1 bar), the reaction yielded ammonia concentrations of up to 82.5 vol% in the reaction mixture.^74^ In the same year, Schüth *et al.*^75^ demonstrated continuous ammonia production over 50 hours using a mechanocatalytic flow setup. An Fe catalyst promoted with cesium (Cs) enabled sustained NH_3_ generation under these conditions. By increasing the Cs content to 4.2 mol% and operating at 20 bar while lowering the reaction temperature, the system maintained stable, continuous ammonia synthesis for nearly 90 hours—representing one of the longest mechanocatalytic gas-phase reactions reported to date. These studies demonstrate that the Cs-promoted iron catalyst has potential for sustained and efficient ammonia production under optimized mechanochemical conditions, though further research on scalability and purification remains necessary. Despite achieving high ammonia concentrations in the reaction mixture, the overall yield remained low (0.44% after 72 hours), indicating that a large fraction of reactants remained unconverted opening space for its recovery and recycling.^75^ While these results show the viability of mechanochemical ammonia synthesis, the method is still in its early stages and not yet suitable for industrial application. The reaction mechanisms are not fully understood, requiring further optimization of reaction time, efficiency, and scalability. Although the energy demand at the laboratory scale is relatively low—about 4.5 × 10^12^ J per ton of NH_3_ compared to 228 × 10^12^ J for the electrified Haber–Bosch process, mechanochemical methods still require substantial development to become industrially viable.^76^

### 3.2 Non-thermal plasma

**Fig. 9:**
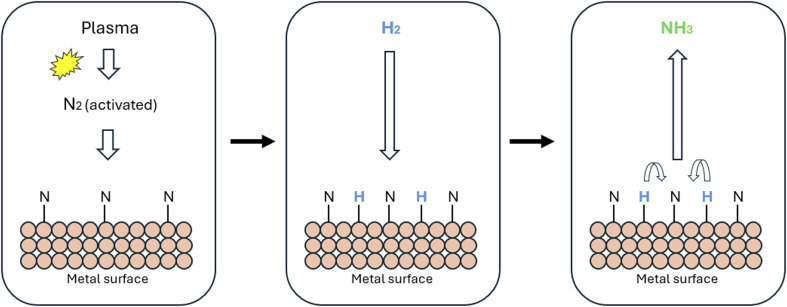
Suggested mechanism for plasma-assisted catalytic ammonia synthesis through plasma induced N_2_ dissociation.^77,79^

Non-thermal plasma (NTP) is a physical method that activates reacting gases by generating partially ionized plasma through the breakdown of gas when an electric field is applied. This high-energy ionized gas promotes chemical reactions, as illustrated in Fig. 9.^77^ A key advantage of NTP is its potential to lower energy consumption and reduce environmental impact compared to traditional processes, which typically require high temperatures (400–500 °C) and pressures (20–40 MPa).^78^ The microwave plasma produces highly energetic electrons capable of activating N_2_ and H_2_, the primary feedstock components for this process. This activation leads to the formation of ions and free radicals, significantly enhancing ammonia formation efficiency. However, NTP technology faces significant challenges: (i) the activation of nitrogen gas due to the cleavage of its strong triple bond (*E*_a_ (N_2_) = 945 kJ mol^−1^) and (ii) the prevention of NH_3_ decomposition. Addressing these issues necessitates developing and selecting catalysts with enhanced plasma-synergistic activities, specifically tailored for relevant plasma conditions.^78,79^ Recent research highlights promising results in catalyst performance for ammonia synthesis. In 2023, NTP using Ru/MgO catalyst, achieved an NH_3_ productivity of 2.67 mmol g_cat_^−1^ h^−1^, with an energy demand of 4.20 kJ L^−1^. Furthermore, innovative methods using seawater as a feedstock yielded results comparable to those achieved with pure H_2_, underscoring the critical role of the Co/SiO_2_ catalyst (energy demand: 3.2 g NH_3_ kW^−1^ h^−1^ at 2 W) in determining NH_3_ productivity.^78,80^ In this approach, H_2_ is generated from seawater *via* electrolysis. Simultaneously, N_2_ and H_2_ are activated using NTP, enabling ammonia synthesis under milder conditions. This method integrates the conventional Haber–Bosch process with plasma activation to improve energy efficiency and environmental sustainability. While NTP represents a promising environmentally friendly approach, the energy demands associated with this process are currently high when compared to the traditional SMR Haber–Bosch process. Experimental data revealed that the NTP process, particularly when integrated with an electrified Haber–Bosch approach, requires approximately 155 GJ of energy, significantly exceeding the 28 GJ needed by the traditional SMR Haber–Bosch method. In summary, substantial challenges in the industrial application of NTP for NH_3_ synthesis persist, including energy demand, scale-up, reactor design, and the stabilization of ammonia to prevent its decomposition. Tackling these issues is crucial for positioning NTP as a viable option for ammonia synthesis.^81^

### 3.3 Electrochemical route

#### 3.3.1 Electrocatalysis

Another significant methodology that has gained importance in recent years is the electrochemical production of green ammonia.^82^ With the anticipated decrease in renewable electricity costs, electrochemical approaches are set to become increasingly viable, offering considerable advantages over traditional methods.^83^ These techniques allow for on-site ammonia synthesis, which not only reduces dependence on centralized production facilities but also minimizes transportation-related CO_2_ emissions, making them an attractive solution for sustainable NH_3_ production.^50^ The fundamental reaction in electrochemical ammonia synthesis involves the reduction of N_2_ to NH_3_ in a catalytic cell, with H_2_ sourced from purified water (H_2_O). At the cathode (Cat.), H_2_O is split to produce H_2_ and electrons (e^−^). At the anode (Anod.), N_2_ is reduced to NH_3_, typically in the presence of a suitable catalyst. This process completes the electrochemical cycle, where the reduction of nitrogen at the anode is coupled with the oxidation/decomposition of water at the cathode, as illustrated by reactions (3.1)–(3.3).^84^ These reactions take place in specialized electrochemical cells that often employ different types of electrolytes. To improve the overall performance of electrochemical ammonia synthesis, various electrolytic technologies can be employed, such as Proton Exchange Membrane (PEM), Anion Exchange Membrane (AEM), and Solid Oxide Electrolysis (SOE), while acknowledging that the intrinsic efficiency of the underlying reactions is limited by thermodynamic constraints. In PEM electrolysis, a solid proton-conducting membrane separates the anode and cathode. This technology is known for its high efficiency, low operating temperatures, and suitability for integration with renewable energy sources due to its ability to efficiently transport protons while minimizing energy losses and enabling rapid response to variable power input. The relevant reactions are provided in eqn (3.1)–(3.3).

3.1 An.: 2H_2_O → O_2_ + 4H^+^ + 4e^−^

3.2 Cat.: N_2_ + 6e^−^ + 6H^+^ → 2NH_3_

3.3 Total: 2N_2_ + 6H_2_O → 4NH_3_ + 3O_2_

PEM technology faces challenges, including the high cost of platinum catalysts and the vulnerability of membranes to contamination and degradation, which can reduce both their efficiency and lifespan. Therefore, continuous research is necessary to develop more cost-effective and durable materials for long-term viability.^85^ AEM electrolysis uses an anion-conducting membrane, which allows for the transport of hydroxide ions (OH^−^). This technology is considered cost-effective because it uses non-precious materials, offering a potential advantage in terms of material costs. Hydrogen production occurs at the cathode, similar to PEM, but with different ion transport mechanisms. The use of non-precious materials offers advantages for AEM technology, but its lower ionic conductivity and membrane instability under alkaline conditions limits efficiency and long-term performance, highlighting the need for ongoing research to improve membrane durability and overall system efficiency.^86^ Finally, SOE systems operate at high temperatures, using a solid oxide electrolyte. They can be highly efficient when paired with heat from renewable sources (such as solar thermal or waste heat) and can directly utilize the heat for water splitting, reducing electricity consumption. However, high operating temperatures also pose challenges, such as material degradation and increased energy requirements for system maintenance, which can impact the long-term viability of SOE systems.^87^ The choice of technology for electrochemical ammonia synthesis depends on multiple factors, with each approach offering unique advantages that must align with the specific process goals and operational conditions. An essential aspect is the coupling with renewable electricity sources, as electrochemical systems can directly operate with intermittent power input. This feature enables the replacement of fossil-based energy supply, thereby reducing CO_2_ emissions while supporting the integration of variable renewable energy into the grid.^88^

#### 3.3.2 Challenges

**Fig. 10:**
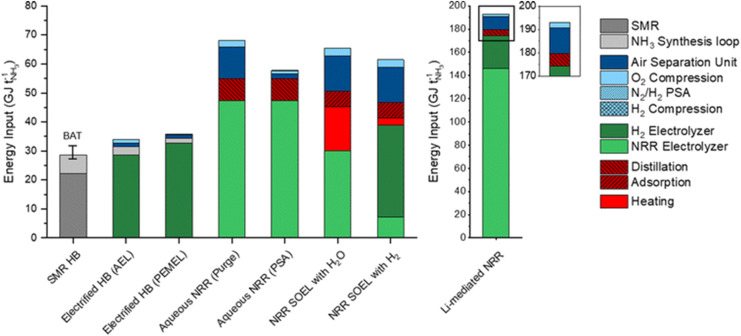
Comparison of the projected energy requirements for conventional Haber–Bosch, electrified Haber–Bosch, aqueous NRR and SOE ammonia synthesis processes.^39^ Reproduced from ref. 39*via* Creative Commons CC BY 3.0 licence

Despite promising advancements, several challenges hinder the commercial realization of electrochemical ammonia synthesis. These include competition with the Hydrogen Evolution Reaction (HER), low N_2_ solubility and activation, catalyst and membrane stability as well as energy efficiency issues. The primary challenge is the competition with HER, which is thermodynamically favoured over the NRR due to its lower energy requirement for H_2_ formation. This competition limits NRR selectivity and reduces NH_3_ yields, making it difficult to reach high production rates.^63^ Overcoming this requires advanced catalyst engineering. Various catalysts, such as transition metals, non-metallics and metal–organic compounds (MOCs), have been explored, but they still fall short of the U.S. Department of Energy's (DOE) performance targets of 7 × 10^−7^ mol cm^−2^ s^−1^ (2520 µmol h^−1^ cm^−2^) at 90% faradaic efficiency.^46,47,89^ Another significant challenge is the low solubility of N_2_ in aqueous media and the high activation energy required for its reduction. In PEM and AEM systems, the limited solubility of N_2_ restricts its availability at the catalyst surface, while the N_2_ triple bond requires substantial energy to break. SOE systems, operating at higher temperatures, improve reaction kinetics but do not fully mitigate this issue. Additionally, the stability of catalysts and membranes is crucial for long-term efficiency. In PEM and AEM systems, catalysts such as Pt and Ir degrade under harsh conditions, while in AEM systems, membrane degradation is exacerbated by chemical corrosion. In SOE systems, high operational temperatures accelerate catalyst degradation. Finally, energy efficiency and system integration remain significant hurdles. In PEM and AEM systems, energy losses arise from membrane resistance and electrode overpotentials, while SOE systems suffer from thermal losses. Overcoming these challenges is vital for the commercialization of electrochemical ammonia production, requiring advancements in catalysts, electrolytes, and system integration.^90–92^ To assess the efficiency of electrochemical ammonia production techniques, it is crucial to consider the various technological approaches employed in these processes. Aqueous NRR and SOE NRR are two prominent methods, each with distinct mechanisms and efficiency profiles. Aqueous NRR typically involves mild conditions and utilizes PEM or AEM membranes, which facilitate ion transport and enable the necessary electrochemical reactions.^93^ These membranes are especially relevant due to their efficient ion conduction in aqueous environments, enhancing overall reaction efficiency. In contrast, SOE NRR leverages thermal energy to drive the reactions, reducing the required electrical energy input.^94^Fig. 10 highlights the significant energy requirements associated with these processes. The traditional Haber–Bosch process requires less than 30 GJ per ton of NH_3_. In comparison, the electrified Haber–Bosch process—where hydrogen is produced *via* water electrolysis using either alkaline (AEL) or proton-exchange membrane (PEMEL) electrolyzers—operates within a similar energy range, between 30–35 GJ per ton of NH_3_. This indicates that while H_2_ production is electrified, the remaining energy demands of the HB process, such as compression and heating, are similar to the conventional process, positioning the electrified route as a competitive alternative. In contrast, direct electrochemical ammonia synthesis *via* aqueous NRR requires significantly higher energy inputs, approximately 50–70 GJ per ton of NH_3_.

This estimate includes energy losses associated with gas purging, product separation, and purification steps such as pressure swing adsorption (PSA), which are necessary to remove unreacted N_2_ and H_2_ and to isolate the produced NH_3_. Despite these elevated energy requirements, NRR using SOE, whether employing water or hydrogen as the reactant, demonstrates comparable energy consumption to the conventional HB process, highlighting the considerable efficiency potential of this technology. Furthermore, smaller energy components, such as heating, distillation, adsorption, O_2_ compression, and air separation, contribute to the overall energy demand, underscoring the importance of optimizing energy use across all stages of the ammonia production process to improve overall efficiency.^39^

#### 3.3.3 Lithium-mediated NRR (Li-mNRR)

The Li-mNRR has garnered significant attention due to its potential for high ammonia production efficiencies. This process utilizes lithium-based electrolytes to facilitate reaction pathways and enhance the catalytic conversion of N_2_ to NH_3_.^68^ This was first explored by Fichter *et al.* in 1930, achieving yields up to 10% at 10 bar.^95^ After a period of stagnation, Tsuneto *et al.*^96^ revitalized the field in 1993, optimizing electrolyte compositions with polar aprotic solvents, leading to faradaic efficiencies (FEs) of 8% at 1 bar and 48% at 50 bar.^84^ In 2020, Lazouski *et al.* introduced a gas diffusion electrode, addressing transport limitations without requiring increased pressure.^97^

Several strategies have since been developed to improve the process, including (I) operating under mild pressure and temperature conditions, (II) introducing small amounts of O_2_, (III) utilizing potential cycling, (IV) using recyclable ionic liquids (ILs) as proton sources, and (V) employing high-surface-area and gas diffusion electrodes.^68^ Despite these advancements, the energy input for Li-mNRR remains high, approximately three times higher than that of aqueous electrochemical NRR or SOEL. The NRR electrolyzer is the primary contributor to this energy demand, with additional energy required for hydrogen electrolyzers, air separation, and oxygen compression. These factors limit the practicality and economic feasibility of the process.^39^ Research efforts are directed toward optimizing Li-based electrolytes to improve electron transport and catalytic NH_3_ formation. While other lithium electrolytes, such as LiPF_6_ and LiTFSI, have been explored for their electrochemical properties, and LiBF_4_ outperforms them in terms of both faradaic efficiency and overall system stability. Additionally, adjusting electrolyte concentration ratios and utilizing aprotic solvents such as tetrahydrofuran (THF) and ethanol (EtOH) enhance N_2_ solubility and accelerate reaction kinetics, leading to increased ammonia yields.^98^ However, Li-mNRR shares challenges with other electrochemical techniques, such as high energy input and suppression of side reactions, notably the HER. HER competes for electrons and protons, reducing ammonia yields and increasing energy consumption. To address this, research is focused on catalyst development to facilitate N_2_ reduction over HER. Advanced materials like transition metal nitrides, borides, and carbides are promising, providing active sites for N_2_ activation while suppressing HER.^46^ A detailed overview and comparison of catalytic systems applied to Li-mNRR is provided in Section 4.2.3.1. Optimizing production rates, faradaic efficiency, costs, and catalyst stability is essential for the practical implementation of Li-mNRR. A notable breakthrough in 2023 by Fu *et al.* achieved a faradaic efficiency of 61% at 1 bar and room temperature in a continuous-flow reactor. Nevertheless, even with 100% faradaic efficiency, the maximum thermodynamic energy efficiency of Li-mNRR is limited to 28%, compared to 63% for the Haber–Bosch process.^67^ This gap highlights the need for further research to improve energy efficiency and make Li-mNRR viable under ambient conditions.

### 3.4 Photo(electro)chemical route

#### 3.4.1 Photocatalysis

**Fig. 11:**
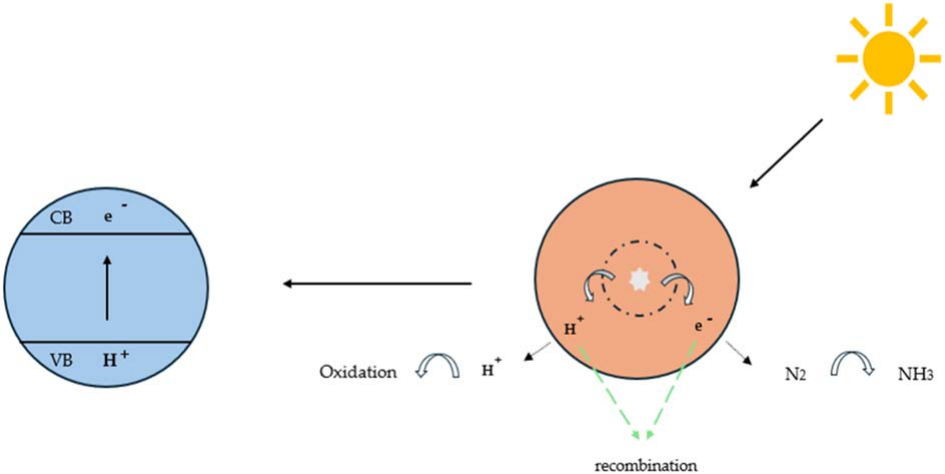
Reaction mechanism of photocatalytic reduction.

Another promising technique that has gained considerable attention in recent years is photo(electro)catalysis, which is employed for the synthesis of various chemical compounds, including NH_3_, H_2_ and CO_2_ conversion products (*e.g.* carbon monoxide, *etc*).^99–101^ This method offers a promising approach for more sustainable synthesis processes, utilizing semiconductor materials that typically absorb light from energy sources such as UV radiation, and in some cases, visible light. Photon excitation initiates reduction and oxidation processes that drive the production of specific species. This generally occurs in three main steps: 1. Photoexcitation, 2. Electron transfer, and 3. Photoreduction & photooxidation. This process can be applied for NRR, where N_2_ is reduced to form NH_3_, starting with photoexcitation, where photons interact with the semiconductor material, causing electrons to be excited from the valence band (VB) to the conduction band (CB). This excitation leaves holes in the VB, which act as oxidative species, while the electrons in the CB function as reductive species. In the following step, the electrons and holes are transferred to the active sites of the photocatalyst and finally photooxidation and photoreduction occur. First, solar-driven H_2_O splitting occurs, where water is dissociated into oxygen and protons, with hydrogen being reduced in the conduction band (CB). Subsequently, nitrogen fixation takes place, where H_2_O molecules are oxidized in the valence band (VB), generating H^+^ and oxygen, and NH_3_ is synthesized as the final product.^48^Fig. 11 illustrates the photocatalytic process, with a particular emphasis on its application to the NRR. There are several challenges associated with the photocatalytic process, with particular emphasis on the recombination of electrons and holes, which can significantly reduce overall efficiency. Recombination can be mitigated by introducing organic scavengers, like methanol (MeOH) or ethanol (EtOH),^102^ which effectively capture intermediate species, preventing their recombination and ensuring a more selective reaction pathway. Well-designed photocatalysts, tailored with specific electronic structures, can also be employed to minimize recombination and enhance efficiency.^103^ Before 2017, faradaic efficiencies were reported below 1%. Yet significant and rapid progress in catalyst design has pushed FE into the 10–20% range, with some cases even surpassing 30%. This highlights the remarkable advancements made in the field, underscoring the importance of continued innovation in catalyst development.^104,105^

#### 3.4.2 Photo electrocatalysis (PEC)

**Fig. 12:**
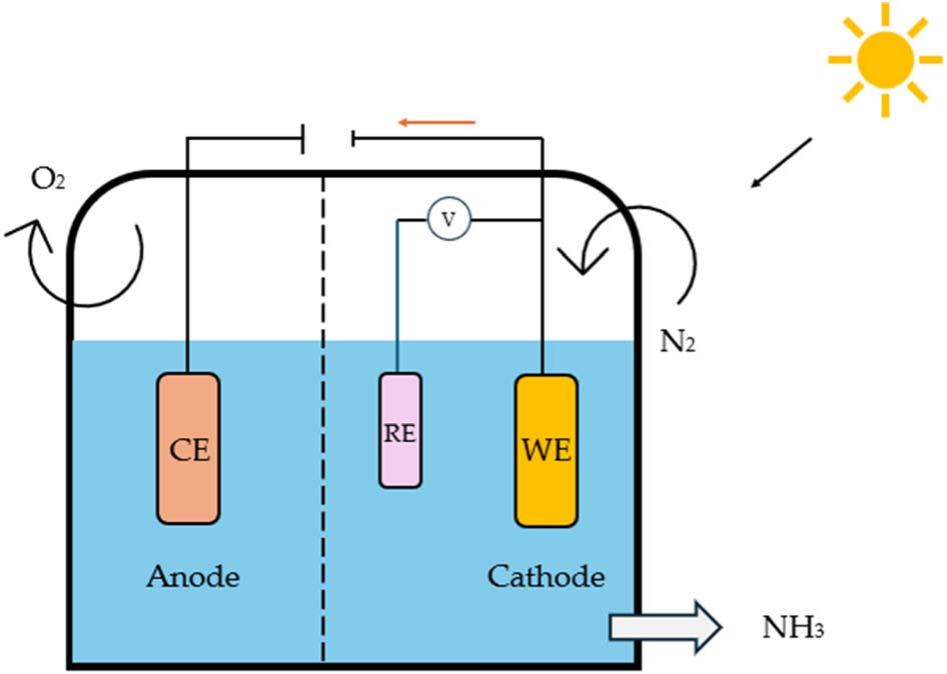
Setup configuration of a photoelectrochemical cell for N_2_ reduction.

The key difference between photocatalysis and photo electrocatalysis lies in how the redox reactions are driven. In photocatalysis, the charge carriers are generated through light absorption, while in photo electrocatalysis, external circuits induce these carriers.^105,106^ By combining the strengths of both approaches—photocatalysis for its light-driven processes and electrocatalysis for its enhanced efficiency, there is significant potential for improvement in sustainability. Despite their promise, both techniques face challenges that often lead to low yields, including high charge carrier recombination, limited light absorption due to mismatched bandgaps, slow electron transfer, and photodegradation.^106–108^ To address these issues, catalyst design plays a pivotal role. Innovations such as doping, cocatalyst deposition, defect integration, and crystal facet tuning can help overcome these barriers.^109^ Furthermore, optimizing the electrolyte to control proton transfer rates and increase N_2_ solubility remains an active area of research.^110^ With continued advancements in catalyst engineering and electrolyte optimization, PEC holds the potential to overcome these current limitations and significantly contribute to more efficient and sustainable ammonia production (Fig. 12).

## 4 Catalyst development and analysis

### 4.1 Towards efficient electrocatalysts for NRR

As highlighted in the previous sections, the demand for efficient electrochemical systems has driven extensive research into effective catalysts, combining theoretical/modelling and experimental efforts. Properties such as crystallinity, morphology, particle size, and the availability of active surface sites are critical for achieving high efficiency and stability. These factors are particularly important under conditions relevant for industrial-scale applications, including extended operation times (>1000 h), moderate temperatures (<100 °C), pressures (∼1 atm), and practical current densities (≥10 mA cm^−2^).^111^ Early studies primarily focused on noble and transition metals, but recent efforts have increasingly explored non-metallic catalysts to overcome the kinetic limitations of the NRR and enhance selectivity under moderate conditions.^63,112^ Metals are often combined with conductive supports (*e.g.*, carbon sheets, graphene oxide), which play a crucial role in catalyst development, aiming to balance efficiency, stability, and cost-effectiveness. A major challenge remains the suppression of the competing HER to maximize NRR efficiency. Since HER often dominates under electrochemical conditions, identifying catalysts with high N_2_ selectivity is essential. Addressing this issue requires a detailed evaluation of catalyst performance based on experimental data or, alternatively, on simulation-based studies based on fundamental principles.^113^ This evaluation faces the challenge of inconsistency and incompleteness of available catalyst data. Reported values for key parameters, such as production rates (PR), faradaic efficiency (FE), and catalyst costs (CC), often lack standardization, making direct comparisons difficult. Although review papers attempt to compile these metrics, discrepancies in measurement conditions and reporting standards persist. To address this, we have developed a structured methodology for the evaluation of the available datasets, highlighting emerging trends, promising catalysts, and identifying critical research gaps.

### 4.2 Methodology for data analysis and graphical representation

To provide a structured and unified approach, a systematic methodology was developed for intuitive data evaluation and visualization. This methodology integrates key metrics, namely PR, FE, and CC, into a cohesive framework for a holistic assessment of catalyst performance and practicality for industrial applications. A major challenge in this analysis was the inconsistency in the reporting of PR, often presented in different units across studies. To standardize the data, production rates were categorized into three units: (1) µmol mg^−1^ h^−1^ (per catalyst mass), (2) µmol cm^−2^ h^−1^ (per electrode area), and (3) µmol h^−1^ L^−1^ g^−1^ (per catalyst mass and reaction medium volume, commonly applied in photocatalytic suspension systems). FE, consistently reported as percentages, were extracted directly from the literature, although the data was limited, particularly for photocatalysts. Despite these gaps, a general comparison of available data was carried out. CC analysis followed a two-tier approach: commercially available catalysts were sourced from chemical suppliers (*e.g.* Sigma-Aldrich) and expressed as cost per gram. Synthesized catalysts were evaluated based on the cost of their raw materials. Whenever possible, bulk pricing was applied to estimate large-scale synthesis costs. A correction factor was applied to refine the cost estimates by accounting for synthesis complexity, material toxicity, and energy demand. Each of these three parameters was evaluated on a scale from 1 to 5, and the correction factor was defined as their arithmetic mean. In all cases, a higher score corresponds to a less favourable characteristic. Synthesis complexity was assessed by examining the reported preparation route, where a score of 1 indicated a simple synthesis and 5 reflected a highly complex procedure. Material toxicity was determined from the safety data sheets (SDS) of the starting materials, with values ranging from 1 (low toxicity) to 5 (high toxicity). Energy demand was evaluated analogously, where 1 represented low energy consumption and 5 denoted highly energy-intensive synthesis conditions. The resulting average score was then multiplied with the raw material cost, providing a more realistic estimate of the overall catalyst cost by incorporating practical synthesis challenges. To enable direct comparison across heterogeneous studies, a scoring system was developed in this work to normalize PR, FE, and CC onto a common scale of 0.5–10 (eqn (4.1)–(4.4)). The lower bound of 0.5 was chosen to avoid assigning zero scores to catalysts with the lowest performance, ensuring all catalysts contribute to the comparative analysis. Higher scores reflect more favourable outcomes, with a maximum combined score of 30.4.1
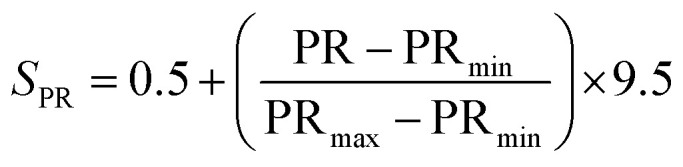
4.2
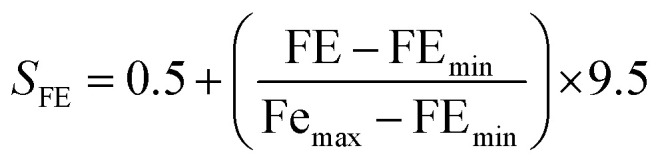
4.3
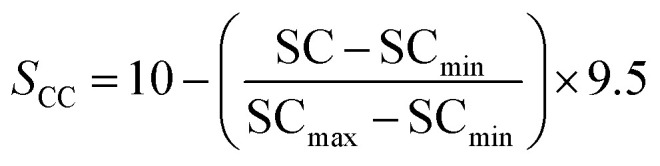

4.4 *S* = *S*_PR_ + *S*_FE_ + *S*_CC_

For the CC analysis, normalization was performed in the same manner, using eqn (4.3), as lower costs are desirable and thus represented by a higher point distribution. To ensure comparability, normalization was performed within each dataset (grouped by methodology and units) relative to the highest value. Data points with exceptionally high values were excluded from the normalization process to prevent distortion. These outliers are highlighted separately in the subsequent analysis to preserve their significance. Critical factors influencing catalyst performance—such as electrolytes, pH, pressure, temperature, and applied potential—are essential for comprehensive analysis. While this information is often incomplete in the literature, it was incorporated where available or noted as missing. Catalytic efficiency is ideally assessed through turnover frequency (TOF), which requires knowledge of the active catalytic surface, often determined using techniques such as SEM or others, such as electrochemical techniques. However, TOF values are rarely reported in the studies analyzed here, which highlights the need for standardized evaluation procedures to enable more comprehensive catalyst benchmarking in the future.

#### 4.2.1 Identification of data-reporting gaps and limitations

As shown in Table 2, ammonia PR are reported using fundamentally different units and normalization strategies, which hinders direct comparison between catalysts. For example, Ni-wire electrodes are reported per area (223 nmol cm^−2^ s^−1^), P-C_3_N_4_ as an absolute rate (4.9 µmol h^−1^), and Co_3_Fe-MOF per mass (8.79 µg h^−1^ mg_cat_^−1^), illustrating how reported differences may reflect normalization rather than intrinsic activity. This inconsistency is pervasive across NH_3_ electrocatalysis and photocatalysis, hindering reliable benchmarking. In related fields such as HER and CO_2_RR, area-normalized rates (µmol cm^−2^ s^−1^) are standard, providing a direct measure of intrinsic activity and enabling cross-study comparison. Mass- and BET-normalized values offer complementary insight for heterogeneous and porous catalysts, reflecting material efficiency and surface effects. For future studies and potential scale-up, adopting area normalization as the primary metric, supplemented by mass and BET data, would establish a consistent and informative framework for evaluating catalytic performance. To address the issue of heterogeneous metrics, the PR values of the 215 catalysts collected in our database were normalized and subsequently evaluated using a structured scoring framework as described in Section 4.2 providing a consistent basis for cross-catalyst comparison.

**Table 2:** Comparison of NH_3_ production-rate normalization and reporting of key catalytic parameters across selected catalytic systems

Entry	Catalyst	Production rate (PR)	Turnover frequency (TOF)	Surface area (ECSA, BET)	Stability	References
12	Ni-wire	223 nmol cm^−2^ s^−1^	Not reported	Not reported	96 h (continuous)	114
14	Mo-foil	0.22 nmol cm^−2^ s^−1^	Not reported	Not reported	Not reported	115
22	Cu	58 nmol cm^−2^ s^−1^	Not reported	Not reported	Not reported	116
123	P-C_3_N_4_	4.9 µmol h^−1^	Not reported	Reported BET	Stability test reported (reusable)	117
				10 m^2^ g^−1^		
199	O-g-C_3_N_4_	118.8 mg L^−1^ h^−1^ g_cat_^−1^	Not reported	Reported BET	20 h (continuous)	118
				220.16 m^2^ g^−1^		
31	V_2_CT_*x*_ Mxene	12.6 mg h^−1^ mg_cat_^−1^	Not reported	Not reported	2 h (cycling); 24 h (continuous)	119
66	Co_3_Fe-MOF	8.79 µg h^−1^ mg_cat_^−1^	Not reported	Reported (ESCA)	2 h (cycling, 4 cycles)	120
				17.74 mF cm^−2^		
155	Pt_1_/N-MoS_2_	121.2 µmol g_cat_^−1^ h^−1^	Not reported	Not reported	5 h (cycling, 4 cycles)	121
159	Cs_2_O/Os–Au	2685 µmol h^−1^ g Os^−1^	Not reported	Not reported	Not reported	122

Building on this, Table 2 highlights further gaps in reporting key catalytic parameters. Most notably, TOF is not reported for the selected systems, limiting assessment of intrinsic activity. Surface-area reporting is inconsistent: O-g-C_3_N_4_ includes BET measurements, P-C_3_N_4_ and Co_3_Fe-MOF report ESCA data, while several systems—including Ni-wire, Mo-foil, Cu, V_2_CT_*x*_ Mxene, Pt_1_/N-MoS_2_, and Cs_2_O/Os–Au—provide none. Stability assessments vary from extended continuous operation (Ni-wire, 96 h) to short-term cycling (V_2_CT_*x*_ Mxene, 2 h; Pt_1_/N-MoS_2_, 5 h), or are entirely omitted (Mo-foil, Cu, Cs_2_O/Os–Au). This combination of inconsistent normalization, missing TOF, variable surface-area data, and heterogeneous stability testing complicates benchmarking and can mislead apparent performance comparisons.

Stability and long-term performance are particularly underreported, yet they are crucial for evaluating catalyst durability and guiding scale-up efforts. Recent Li-mNRR studies have demonstrated that *operando* and *in situ* characterization techniques—such as GI-WAXS,^123^*in situ* ATR-SEIRAS,^124^ and flow-cell X-ray scattering^125^—provide time-resolved insights into the evolution of the solid-electrolyte interphase, surface intermediates, and dynamic structural changes under reaction conditions. These approaches allow researchers to directly correlate structural and chemical changes with catalytic performance, enabling systematic evaluation of stability, understanding of deactivation mechanisms, and guidance for the design of more robust catalysts.

Adopting a standardized reporting framework, including TOF, surface area, stability, and consistent normalization, would enable more rigorous comparisons and clearer insights into intrinsic catalytic activity.

As indicated by our data analysis of catalytic systems (Tables 6–8), photocatalytic nitrogen reduction remains comparatively underrepresented relative to electrocatalytic and Li-mediated approaches. Many commonly employed materials—such as transition-metal centers (Mo, Ni, Cu), high-surface-area scaffolds (MXenes, MOFs), and defect-engineered heterostructures—are intrinsically photoactive, yet they are predominantly explored under electrochemical bias or in Li-mediated systems. This is exemplified by MXene–metal oxide heterostructures, which are primarily designed to maximize charge transfer kinetics, surface adsorption, and defect-mediated activity under applied potential, rather than optimizing light absorption or photogenerated carrier separation.^126^ As highlighted by Ranjith *et al.*, this underrepresentation reflects the intrinsic challenges of photo-driven N_2_ activation.^127^ Developing efficient heterojunction photocatalysts remains difficult due to low conversion efficiencies and strong material dependence, requiring simultaneous optimization of light absorption, charge separation, band alignment, and redox potential. Rapid recombination of photogenerated carriers, limited visible-light harvesting, competitive hydrogen evolution, and insufficient surface electron density in aqueous media further restrict activity.^128^ Consequently, advanced heterostructures rarely achieve measurable ammonia formation under purely photocatalytic conditions, explaining the sparse and heterogeneous representation and the difficulty of generating reproducible, reportable data.

#### 4.2.2 Integration of catalyst cost into performance assessment

**Fig. 13:**
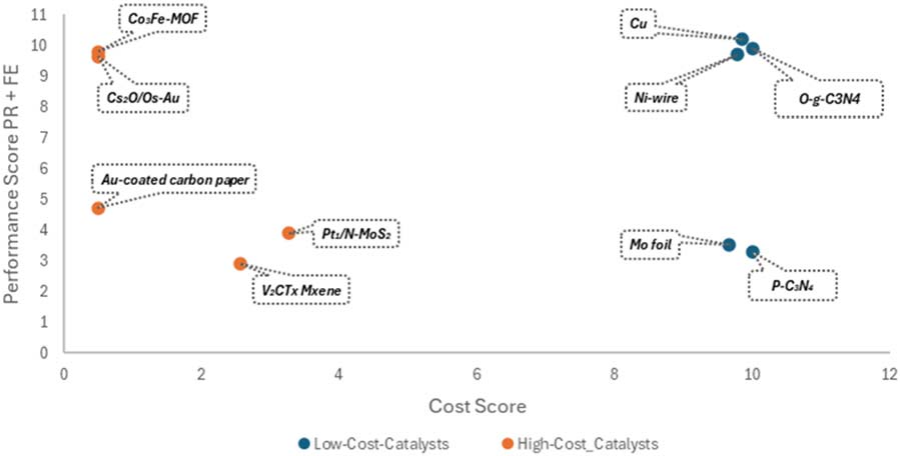
Cost performance comparison of selected catalysts.

To highlight the novel cost-performance evaluation framework developed in this study, an illustrative analysis was conducted prior to the detailed discussion of individual catalytic systems. Ten representative catalysts spanning electrochemical NRR (electrocatalysis), lithium-mediated NRR (Li-mNRR), and photoelectrocatalysis were selected based on their contrasting cost characteristics, categorized as low-cost and high-cost materials. The resulting visualization (Fig. 13) demonstrates how the combined consideration of production rates, faradaic efficiency, and catalyst cost can guide the identification of promising catalyst families for further research and scale-up. Non-noble metals (*e.g.*, Mo-, Ni-, and Cu-based catalysts) generally exhibit a favorable cost-to-performance ratio compared with noble-metal-based materials, while synthesis complexity further modulates the overall cost. This approach underscores the practical relevance of integrating economic considerations alongside performance metrics and provides a clear, actionable overview ahead of the comprehensive analysis of all 215 catalytic systems. Details for the remaining catalysts, including their performance and cost metrics, are provided in the SI (Table S1), with the original literature sources of each catalyst explicitly cited.

#### 4.2.3 Results and interpretation of catalyst performance evaluation

**Fig. 14:**
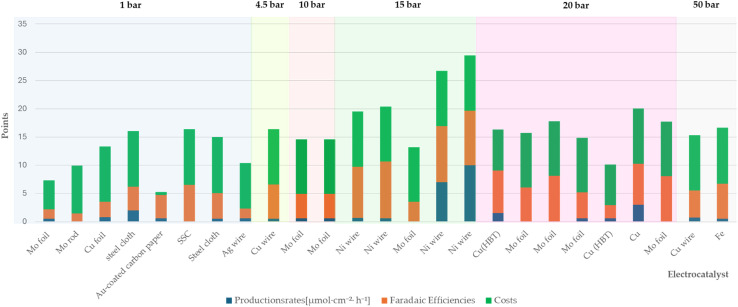
Comparison of catalysts for the Li-mNRR.

**Fig. 15:**
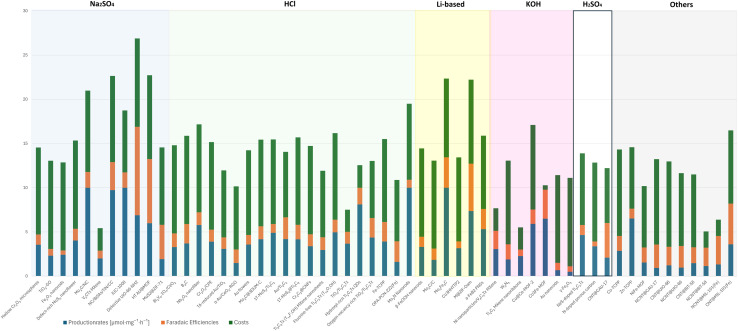
Comparative analysis of diverse electrocatalysts in varied electrolyte compositions.

**Fig. 16:**
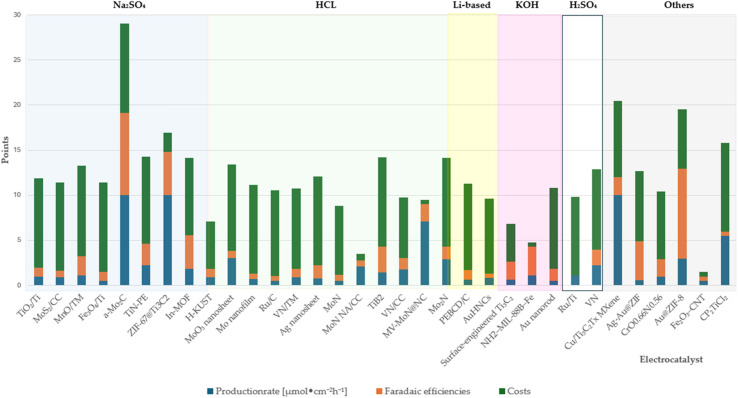
Comparative analysis of electrocatalysts in varied electrolyte compositions.

**Fig. 17:**
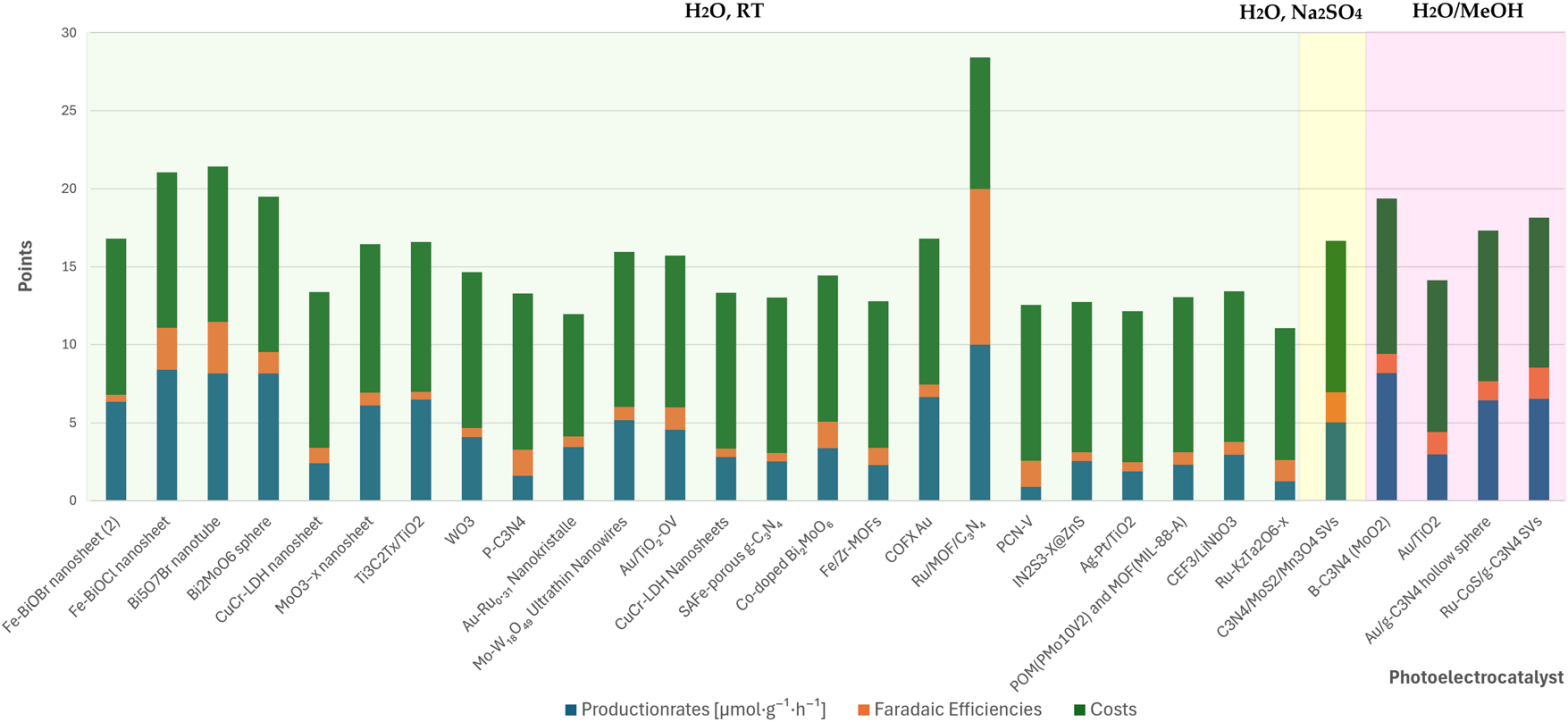
Performance comparison of different photocatalytic systems.

**Fig. 18:**
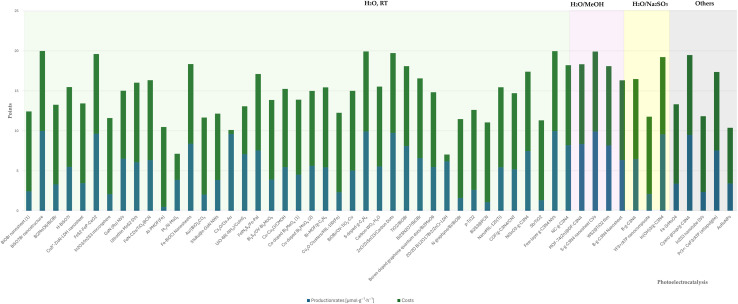
Performance comparison of different photocatalytic systems.

**Fig. 19:**
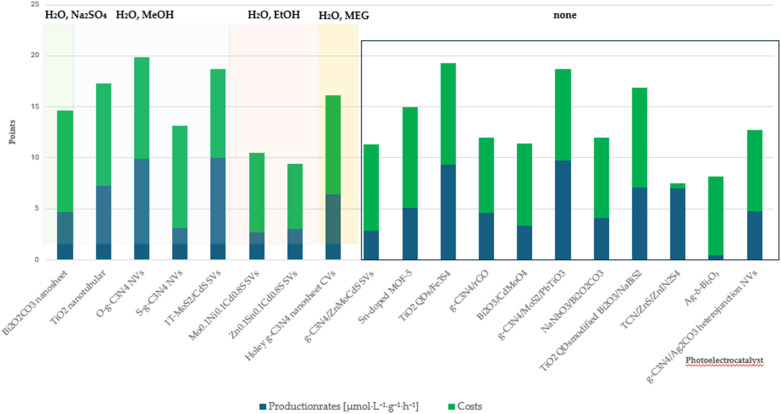
Comparison of photo electrocatalysts.

This section presents the results of the catalyst performance evaluation for Li-mNRR and (photo-)electrocatalysis. Based on the metrics of production rates, faradaic efficiency, and catalyst costs, a comparative analysis of the various catalytic systems was conducted to identify performance trends and trade-offs (Fig. 14–19) visualize the point distribution of the assessed catalysts for each technique, as determined through eqn (4.1)–(4.3). The *x*-axis shows the tested catalysts, while the *y*-axis represents the total score, *S*, signalling the contribution of each criterion in different colours: blue (*S*_PR_), orange (*S*_FE_), and green (*S*_CC_). The background colours in the diagrams provide additional context, regarding the applied pressure for Li-mNRR (Fig. 14) and the electrolytes for (photo-)electrocatalysis (Fig. 15–19). This approach allows for an easy and quantitative comparison of the catalysts' performance across different conditions and media.

##### 4.2.3.1 Li-mediated nitrogen reduction reaction (Li-mNRR)

The literature data showed that nickel, molybdenum and copper are widely used catalysts, although other species have also been studied (*e.g.* steel, Ag, Fe), as summarized in Table 3. Nickel wire under elevated pressure (15 bar) showed the highest performance (*S* = 29.4), as shown in Fig. 14. This is likely due to nickel's electronic properties, which facilitate efficient electron transfer, and its strong interaction with N_2_, as indicated by the high binding energy of the Ni(N_2_)_4_ complex (120 kJ mol^−1^). Both aspects contribute to effective nitrogen activation.^129^ Molybdenum exhibits modest ammonia production rates at atmospheric pressure (1 atm) and lacks comprehensive data for elevated pressures (15 and 20 bar), molybdenum remains a compelling candidate due to its strong affinity for nitrogen binding and its versatile oxidation states.^130,131^ Notably, molybdenum is a key component in the active sites of different enzymes, such as nitrogenase, found in nature, in the pathway of nitrogen fixation, which efficiently catalyse nitrogen-based molecules reduction. These properties contribute to moderate the high faradaic efficiencies in molybdenum-based catalysts, particularly under increased pressure. Furthermore, molybdenum's relative abundance and cost-effectiveness enhance its appeal for industrial applications. As of early 2025, the price of high-purity molybdenum ranges between €25 and €90 per kilogram, depending on form and purity levels.^132^ Copper has shown considerable promise, particularly at atmospheric pressure, where its ammonia production rates are comparable to those of molybdenum, with further improvements observed under elevated pressure conditions. The copper-based compound Cu(HBT) demonstrates high faradaic efficiency, elevated production rates, and moderate costs, highlighting the influence of ligands on enhancing copper's catalytic performance. Copper's high faradaic efficiencies are largely attributed to its excellent electrical conductivity (∼5.96 × 10^7^ S m^−1^ at 20 °C) which boosts catalytic performance.^133^ As of early 2025, the price of high-purity copper ranges between €6 and €9 per kilogram, depending on form and purity levels, making it an economically attractive starting material for industrial applications. Alternative materials, such as Au-coated carbon paper, Ag, Fe, and stainless-steel cloth (SSC), have been investigated for their catalytic potential in Li-mNRR, but their overall performance generally falls short in comparison with Ni, Mo and Cu. Nevertheless, SSC stands out within this group due to its favourable combination of moderate ammonia production rates, appreciable faradaic efficiencies, and low material costs, making it a potentially scalable option for industrial applications. Beyond catalyst composition, the choice of electrolyte exerts a substantial influence on reaction kinetics and overall NRR efficiency. Among the lithium-based electrolytes, LiBF_4_ has demonstrated superior performance, with reported FE's reaching up to 61%.^98^ In particular, synergistic effects have been observed when LiBF_4_ is paired with copper-based catalysts, where electrolyte-catalyst interactions appear to enhance N_2_ activation and electron transfer efficiency. These findings highlight the critical importance of electrolyte-catalyst coupling in optimizing Li-mNRR systems. Continued refinement of catalyst materials—especially Ni, Mo, and Cu—when used in conjunction with high-performance electrolytes such as LiBF_4_, could lead to substantial improvements in efficiency, scalability, and cost-effectiveness. This approach holds significant promise for the development of viable technologies for sustainable, industrial-scale ammonia synthesis.

**Table 3:** Evaluation scores of various electrode materials based on NH_3_ production rate, faradaic efficiency, and estimated catalyst cost

Entry	Catalyst	Score (points)	References
1	Mo foil	7.3	115
2	Mo rod	9.9	134
3	Cu foil	13.3	135
4	Steel cloth	16.0	136
5	Au-coated carbon paper	5.2	137
6	SSC	16.4	138
7	Steel cloth	15.0	139
8	Ag wire	10.4	140
9	Cu wire	16.4	141
10	Mo foil	14.6	142
11	Mo foil	14.6	143
12	Ni-wire	19.5	114
13	Ni-wire	20.4	114
14	Mo-foil	13.2	144
15	Ni wire	26.7	145
16	Ni wire	29.4	145
17	Cu(HBT)	16.3	146
18	Mo-foil	15.7	144
19	Mo-foil	17.8	144
20	Mo-foil	14.8	144
21	Cu(HBT)	10.1	147
22	Cu	20.1	148
23	Mo-foil	17.7	144
24	Cu wire	15.3	149
25	Fe	16.6	96

##### 4.2.3.2 Electrocatalysis

###### 4.2.3.2.1 Production rates per mass of catalyst ([µmol mg^−1^ h^−1^])

The evaluated data, reported in Fig. 15 and summarized in Table 4, reveal that molybdenum-based compounds—such as Mo_2_N nanorods, MoS_2_, and Mo_3_Fe_3_C—consistently achieve high FEs and notable ammonia PR, while maintaining moderate material costs. In addition to these intrinsic properties, the choice of electrolyte—ranging from acidic to basic—also contributes to the observed catalytic performance and selectivity. Building on the favourable properties discussed previously, their catalytic performance is further enhanced when molybdenum is combined with elements such as carbon, nitrogen, or sulphur.^131,150–152^ These heteroelemental combinations introduce electronic modifications, alter the d-band structure, and promote the formation of vacancy-rich or defect-engineered sites, which collectively improve N_2_ activation and facilitate multi-electron transfer processes. The synergy between intrinsic material properties and structural tunability makes molybdenum-based systems highly adaptable for nitrogen reduction applications and supports their growing prominence in the field. MOFs, TCPP-based ligands, and MXenes demonstrate outstanding catalytic performance in electrochemical NRR, characterized by high faradaic efficiencies and moderate to high ammonia production rates. Their superior activity is primarily attributed to their intrinsic porosity, which provides a high density of accessible active sites and promotes efficient mass and charge transport.^153^ Furthermore, the tuneable chemical composition and structural flexibility of these materials allow for precise modulation of their electronic and catalytic properties. Despite their promising performance, the relatively high synthesis costs and limited scalability of these materials currently hinder their widespread application.^154,155^ To address these challenges, defect engineering has emerged as a complementary strategy to enhance catalytic activity. By introducing vacancy sites or structural irregularities, materials such as defective UiO-66-NH_2_ and defect-rich MoS_2_ nanoflowers exhibit improved N_2_ adsorption and activation, thereby lowering the energy barrier for ammonia synthesis.^156^ The combined benefits of structural tunability and defect engineering underscore the potential of these advanced materials for high-performance nitrogen reduction. These findings are summarized in Fig. 15, which highlights the correlation between material class, structural characteristics, and catalytic performance.

**Table 4:** Evaluation scores of various electrode materials based on NH_3_ production rate, faradaic efficiency, and estimated catalyst cost

Entry	Catalyst	Score (points)	References
26	Hollow Cr_2_O_3_ microspheres	14.6	157
27	TiO_2_-GO	13.1	158
28	Fe_2_O_3_ nanorods	12.9	159
29	Defect-rich MoS_2_ nanoflower	15.3	160
30	Mo_2_C/NC	21.0	161
31	V_2_CT_*x*_ MXene	5.4	162
32	NC/BiSAs/TiN/CC	22.6	163
33	JUC-1000	18.7	164
34	Defective UiO-66-NH_2_	26.9	165
35	HT-Au@MOF	22.7	166
36	MoS2@ZIF-71	14.6	167
37	Bi_2_V_0_.1O_*x*_/CeO_2_	14.8	168
38	B_4_C	15.9	169
39	Nb_2_O_5_ nanofiber	17.2	170
40	Cr_2_O_3_/CPE	15.2	171
41	TA-reduced Au/TiO_2_	12.0	172
42	α-Au/CeO_2_-RGO	10.2	173
43	Au flowers	14.2	174
44	Mo_2_C@3DUM-C	15.4	175
45	Au/Ti_3_C_2_	14.1	176
46	1T-MoS_2_/Ti_3_C_2_	15.5	177
47	Cr_3_C_2_@CNFs	14.7	178
48	Ti_3_C_2_T_*x*_ (T_14_F,OH) MXene nanosheets	11.9	179
49	Fluorine-free Ti_3_C_2_T_*x*_ (T_14_O,OH)	16.2	180
50	TiO_2_/Ti_3_C_2_T_*x*_	7.5	181
51	Hydroxyl-rich Ti_3_C_2_T_*x*_ QDs	12.6	182
52	Oxygen-vacancy-rich TiO_2_/Ti_3_C_2_T_*x*_	13.0	183
53	Fe-TCPP	15.5	184
54	OPA-PCN-222 (Fe)	10.9	185
55	Mo_2_N nanorods	19.5	186
56	β-FeOOH nanorods	14.5	187
57	Mo_2_C/C	13.1	188
58	Mo_3_Fe_3_C	22.3	188
59	Co3HHTTP2	13.4	189
60	M@ZIF-Oam	22.2	190
61	a-FeB_2_ PNSs	15.9	191
62	Ni nanoparticles/V_4_C_3_T_*x*_ MXene	7.7	192
63	W_2_N_3_	13.1	193
64	Ti_3_C_2_ MXene nanoribbons	5.5	194
65	Cu@Ce-MOF-2	17.1	195
66	Co3Fe-MOF	10.3	120
67	Au nanorods	11.4	196
68	γ-Fe_2_O_3_	11.1	197
69	NeS-doped Ti_3_C_2_T_*x*_	13.9	198
70	N-doped porous carbon	12.8	199
71	CNT@CAU-17	12.2	200
72	Co-TCPP	14.3	201
73	Zn-TCPP	14.6	201
74	NiFe-MOF	10.2	202
75	NCNT@CAU-17	13.2	203
76	CNT@UIO-66	13.0	204
77	NCNT@UiO-66	11.7	204
78	CNT@BIT-58	11.5	205
79	NCNT@BIT-58	5.1	205
80	NCNT@MIL-101(Fe)	6.6	206
81	CNT@MIL-101(Fe)	16.5	206

###### 4.2.3.2.2 Production rates per unit area of catalyst [µmol cm^−2^ h^−1^]

Further analysis of the evaluated systems confirms the recurrent effectiveness of molybdenum-based materials (Table 5). Beyond the previously discussed compounds, MoO_3_ nanosheets and Mo_2_C@3DUM-C have demonstrated substantial activity toward nitrogen reduction, reinforcing molybdenum's relevance across various structural forms. In parallel, composite and hybrid materials such as MXene/ZIF systems—including Cu/Ti_3_C_2_T_*x*_, ZIF-67@Ti_3_C_2_, Ag–Au@ZIF, and Au@ZIF-8—as well as MOFs like In-MOF and HKUST-1, exhibit high faradaic efficiencies and significant ammonia production rates, as summarized in Fig. 16. These results underscore the potential of framework-based and two-dimensional materials for NRR applications. A noteworthy organometallic compound in this context is Cp_2_TiCl_2_ (ferrocene analogue), which achieves high ammonia production rates despites its relatively low FE. This discrepancy suggests that while the compound exhibits intrinsic N_2_ reduction activity, its electron transfer efficiency may be insufficient. A potential pathway to improve its performance lies in the integration with highly conductive materials—such as carbon-based nanostructures or MXenes—which may enhance charge transport and stabilize intermediate species. Surface-engineered titanium-based materials also show promise. In particular, Ti_3_C_2_ MXenes containing engineered surface defects demonstrate enhanced activity, likely due to the formation of additional catalytically active sites. Titanium's widespread use is further justified by its corrosion resistance, structural stability, and capacity to form synergistic composites. However, as illustrated in Fig. 16, the performance of Ti-based catalysts varies significantly. While some systems yield promising results, others remain less effective, indicating that the catalytic behaviour is highly sensitive to structural and compositional factors. Furthermore, current data on Ru/Ti systems remain incomplete—particularly with respect to faradaic efficiency—which hinders full performance evaluation and comparison. Finally, no clear correlation has yet emerged between the choice of electrolyte and catalytic output, highlighting a knowledge gap requiring systematic investigation in future studies.

**Table 5:** Evaluation scores of various electrode materials based on NH_3_ production rate, faradaic efficiency, and estimated catalyst cost

Entry	Catalyst	Score (points)	References
82	TiO_2_/Ti	14.6	207
83	MoS_2_/CC	13.1	208
84	MnO/TM	12.9	187
85	Fe_3_O_4_/Ti	15.3	209
86	a-Mo_2_C	**21.0**	210
87	TiN-PE	5.4	211
88	ZIF-67@Ti_3_C_2_	22.6	212
89	In-MOF	18.7	213
90	H-KUST	26.9	214
91	MoO_3_ nanosheet	22.7	215
92	Mo nanofilm	14.6	216
93	Ru/C	14.	217
94	VN/TM	15.9	218
95	Ag nanosheet	17.2	219
96	MoN	15.2	220
97	MoN NA/CC	12.0	221
98	TiB2	10.2	222
99	VN/CC	14.2	223
100	MV-MoN@NC	15.4	224
101	Mo_2_N	14.1	47
102	PEBCD/C	15.5	225
103	AuHNCs	14.7	226
104	Surface-engineered Ti_3_C_2_	11.9	46
105	NH_2_-MIL-88B-Fe	16.2	227
106	Au-nanorod	7.5	228
107	Ru/Ti	12.6	229
108	VN	13.0	230
109	Cu/Ti_3_C_2_T_*x*_ MXene	15.5	231
110	Ag–Au@ZIF	10.9	232
111	CrO_0.66_N_0.56_	19.5	233
112	Au@ZIF-8	14.5	234
113	Fe_2_O_3_-CNT	13.1	47
114	CP_2_TiCl_2_	22.3	235

###### 4.2.3.2.3 Photocatalysis

The comparison of various photocatalysts is based on reported experiments conducted under moderate temperatures, as summarized in Tables 6 and 7. In many cases, the authors specified temperatures within the range of 15–30 °C, which in this work is generalized as room temperature. For clarity, the graphical representation is divided into two figures: Fig. 17 summarizes systems for which both production rates and faradaic efficiencies were reported, whereas Fig. 18 presents data from studies in which faradaic efficiencies were not available. Although the results for photocatalysts exhibit limited variability overall, a few systems clearly stand out—most notably Ru/MOF/C_3_N_4_, P-C_3_N_4_, and Bi_5_O_7_Br nanotubes. While these materials demonstrate superior performance, the majority of photocatalysts show rather uniform behavior in terms of production rates, faradaic efficiencies, and costs. A particularly interesting observation is that the most efficient catalysts often share structural and electronic characteristics, including optimized bandgaps and enhanced charge carrier separation. A consistent trend emerges with graphitic carbon nitride (g-C_3_N_4_): its high photocatalytic activity is commonly attributed to its narrow bandgap (∼2.7 eV), enabling visible light absorption, and to its excellent thermal and chemical stability under harsh conditions. Furthermore, its layered structure provides a high specific surface area, facilitating effective adsorption of reactants and improving reaction kinetics.^236^ As a result, g-C_3_N_4_ is frequently modified or combined with other materials—such as molybdenum compounds (*e.g.*, MoO_2_), gold (Au), MOFs, or ruthenium (Ru)—to enhance its photocatalytic performance. Notably, the integration of noble metals or the construction of heterojunctions has been shown to improve charge carrier dynamics by promoting charge separation and reducing recombination losses. These effects are often realized through the formation of internal electric fields or Schottky junctions at the material interfaces, both of which facilitate unidirectional charge flow and enhance photocatalytic efficiency. As is well-documented in the literature, doping and defect engineering are additional effective strategies to modulate the electronic structure and surface reactivity. These modifications allow for fine-tuning of band positions, increased density of active sites, and improved kinetics of key reaction steps, such as N_2_ adsorption and activation. Bismuth-based materials also show strong potential, with compounds like Bi_5_O_7_Br demonstrating higher faradaic efficiencies and production rates compared to many alternatives. Their favorable bandgaps (typically 1.8–2.8 eV) support efficient visible light absorption, while their chemical flexibility and stability in aqueous environments make them suitable for surface and structural tailoring, such as through doping or nanoscale morphology control, to further optimize their catalytic behavior.^237,238^ Moreover, materials previously studied as electrocatalysts, including molybdenum compounds, TiO_2_, Ti_3_C_2_ MXenes, MOF, and other porous structures, are increasingly utilized in photocatalysis, owing to their high surface areas, stability, and intrinsic catalytic activity.^239^ While Fig. 16 provides a comparative overview of photocatalyst performance, it does not conclusively indicate whether variations in electrolytes significantly impact the reaction mechanism and further studies are needed to shed light on this aspect. Fig. 17 presents ammonia production rates for selected materials under mild conditions (15–30 °C) along with their electrolyte compositions. Even though faradaic efficiency data are not available for these experiments, production rates alone offer valuable insights. Materials such as C_3_N_4_, bismuth-based compounds, MOFs, and TiO_2_ demonstrate notably high ammonia yields, highlighting their potential for nitrogen reduction under ambient conditions. Their favorable structural and electronic properties, such as optimized bandgaps and high surface areas, facilitate efficient N_2_ activation upon light exposure, enhancing overall photocatalytic performance. No clear correlation between electrolyte composition and catalytic output was observed, and variations within the tested temperature range appear minimal, suggesting that neither factor strongly dominates under these conditions.

**Table 6:** Evaluation scores of various electrode materials based on NH_3_ production rate, faradaic efficiency, and estimated catalyst cost

Entry	Catalyst	Score (points)	References
115	Fe-BiOBr nanosheet (1)	16.8	240
116	Fe-BiOCl nanosheet (2)	21.1	241
117	Bi_5_O_7_Br nanotube	21.4	242
118	Bi_2_MoO_6_ sphere	19.5	243
119	CuCr-LDH nanosheet	13.4	244
120	MoO_3−*x*_ nanosheet	16.4	245
121	Ti_3_C_2_T_*x*_/TiO_2_	16.6	246
122	WO_3_	14.7	247
123	P-C_3_N_4_	13.3	248
124	Au–Ru_0_._31_ nanokristalle	12.0	249
125	Mo-W_18_O_49_ ultrathin nanowires	16.0	250
126	Au/TiO_2_-OV	15.7	251
127	CuCr-LDH nanosheets	13.3	252
128	SAFe-porous g-C_3_N_4_	13.0	253
129	Co-doped Bi_2_MoO_6_	14.4	254
130	Fe/Zr-MOFs	12.8	255
131	COFX Au	16.8	256
132	Ru/MOF/C_3_N_4_	28.4	257
133	PCN-V	12.5	258
134	IN_2_S_3_-X@ZnS	12.7	259
135	Ag–Pt/TiO_2_	12.2	260
136	POM(PMo_10_V_2_) and MOF(MIL-88-A)	13.0	261
137	CEF_3_/LiNbO_3_	13.4	262
138	Ru-KzTa_2_O_6−*x*_	11.1	263
139	C_3_N_4_/MoS_2_/Mn_3_O_4_ SVs	16.7	264
140	B-C_3_N_4_ (MoO_2_)	19.4	265
141	Au/TiO_2_	14.1	266
142	Au/g-C_3_N_4_ hollow sphere	17.3	267
143	Ru-CoS/g-C_3_N_4_ SVs	18.1	268

**Table 7:** Evaluation scores of photocatalysts based on NH_3_ production rate, faradaic efficiency, and estimated catalyst cost

Entry	Catalyst	Score (points)	References
144	BiOBr nanosheet (1)	12.4	269
145	Bi_5_O_7_Br nanostructure	20.0	270
146	Bi_2_MoO_6_/BiOBr	13.3	271
147	H-Bi_5_O_7_I	15.5	272
148	Cuδ^+^-ZnAl-LDH nanosheet	13.4	273
149	FeS_2_-FeP-CeO_2_	19.6	274
150	In_2_O_3_/In_2_S_3_ microsphere	11.6	275
151	GaN (Ru) NVs	15.0	276
152	Ultrathin MoS_2_ SVs	16.0	277
153	FeN-CDs/TiO_2_@CN	16.3	278
154	Al-PMOF(Fe)	10.5	279
155	Pt_1_/N-MoS_2_	7.1	280
156	Fe-BiOCl nanosheets	18.4	281
157	Au/(BiO)_2_CO_3_	11.7	282
158	5%Ru@n-GaN NWs	12.2	283
159	Cs_2_O/Os–Au	10.1	284
160	UiO-66(–NH_2_)/CuInS_2_	13.1	285
161	FeIN_2_S_4_/Fe-Pal	17.1	286
162	Bi_2_S_3_/OV-Bi_2_MoO_6_	13.9	287
163	Cu–Cu_2_O/CMOH	15.3	288
164	Co-doped Bi_2_MoO_6_ (1)	13.9	289
165	Cu-doped Bi_2_MoO_6_ (2)	15.0	290
166	Bi-MOF/g-C_3_N_4_	15.4	291
167	Cu_2_O clusters/MIL-100(Fe)	12.3	292
168	BiOBr/OV-TiO_2_-Cu	15.0	293
169	S-doped-g-C_3_N_4_	19.9	294
170	Carbon-WO_3_·H_2_O	15.5	295
171	ZnO/ZnSnO_3_/carbon dots	19.7	296
172	TiO_2_/BiOBr	18.1	297
173	Bi_2_SN_2_O_7_/BiOBr	16.6	298
174	Boron-doped graphene quantum dots/Bi_2_MoO_6_	14.8	299
175	2D/2D Bi_12_O_17_Br_2_/ZnCr-LDH	7.0	300
176	N-graphyne/Bi/BiOBr	11.5	301
177	p-TiO_2_	12.6	302
178	Bi_2_S_3_@PCN	11.1	303
179	NanoMIL-125(Ti)	15.5	304
180	COF/g-C_3_N_4_/CNT	14.7	305
181	NiSnO_3_-g-C_3_N_4_	17.4	306
182	Sb/TiO_2_	11.3	307
183	Few-layer g-C_3_N_4_ NVs	20.0	308
184	NC-g-C_3_N_4_	18.2	309
185	MOF-74(Zn)@DF-C_3_N_4_	18.3	310
186	S-g-C_3_N_4_ nanosheet CVs	19.9	311
187	WS_2_@TiO_2_ film	18.1	312
188	B-g-C_3_N_4_ nanosheet	16.3	313
189	B-g-C_3_N_4_	16.5	314
190	YF3+/ATP nanocomposite	11.8	315
191	In(OH)_3_/g-C_3_N_4_	19.2	316
192	Fe-SrMoO_4_	13.3	317
193	Cyano group/g-C_3_N_4_	19.5	318
194	In_2_S_3_ nanotube SVs	11.8	319
195	Pr^3+^:CeF_3_/ATP (attapulgite)	17.4	320
196	AuRuNPs	10.4	321

###### 4.2.3.2.4 PEC performance [µmol mg^−1^ L^−1^ h^−1^]

A comparative overview of photocatalyst performance in flow systems, expressed in µmol mg^−1^ L^−1^ h^−1^, is provided in Fig. 19, while the corresponding data are summarized in Table 8. Although quantum efficiency values are not reported in the dataset, the catalytic systems are represented by their production rates and estimated costs. Among the evaluated systems, the most active materials include O-g-C_3_N_4_ NVs, C_3_N_4_-MoS_2_-PbTiO_3_, and TiO_2_ modified with Bi_2_O_3_/NaBiS_2_ as well TiO_2_ QDs/FeS_4_. A recurring pattern is observed across the most efficient systems: many incorporate elements such as molybdenum, TiO_2_, g-C_3_N_4_, or bismuth, either as individual phases or within composite structures. These components appear to play a central role in enhancing light absorption, improving charge separation, and promoting N_2_ activation, thereby driving photocatalytic performance. Although the subsequent data section does not include faradaic efficiency values, the observed production rate trends remain consistent with previous findings. Regarding solvent effects, mixed solvent systems such as H_2_O/MeOH or H_2_O/EtOH do not reveal a clear trend, suggesting that solvent influence is likely system-specific and should be addressed in more targeted investigations.

**Table 8:** Evaluation scores of photo electrocatalysts based on NH_3_ production rate, faradaic efficiency, and estimated catalyst cost

Entry	Catalyst	Score (points)	References
197	Bi_2_O_2_CO_3_ nanosheet	14.6	322
198	TiO_2_ nanotubular	17.3	323
199	O-g-C_3_N_4_ NVs	19.9	324
200	S-g-C_3_N_4_ NVs	13.1	324
201	1T-MoS_2_/CdS SVs	18.7	325
202	Mo_0.1_Ni_0.1_Cd_0.8_S SVs	10.5	326
203	Zn_0.1_Sn_0.1_Cd_0.8_S SVs	9.4	327
204	Holey g-C_3_N_4_ nanosheet CVs	16.2	328
205	g-C_3_N_4_/ZnMoCdS SVs	11.3	329
206	Sn-doped MOF-5	14.9	330
207	TiO_2_ QDs/Fe_3_S_4_	19.3	331
208	g-C_3_N_4_/rGO	12.0	332
209	Bi_2_O_3_/CdMoO_4_	11.4	333
210	g-C_3_N_4_/MoS_2_/PbTiO_3_	18.7	334
211	NaNbO_3_/Bi_2_O_2_CO_3_	12.0	335
212	TiO_2_ QDsmodified Bi_2_O_3_/NaBiS_2_	16.9	336
213	TCN/ZnS/ZnIN_2_S_4_	7.5	337
214	Ag-δ-Bi_2_O_3_	8.2	338
215	g-C_3_N_4_/Ag_2_CO_3_ heterojunction NVs	12.8	339

### 4.3 Machine learning (*k*-means clustering) catalyst analysis for eNRR

Machine learning (ML) emerges as a powerful tool in several areas of activity. Recently it has been explored with focus on catalyst discovery and optimization, offering the potential to significantly accelerate research in electrochemical ammonia synthesis.^340,341^ Traditional experimental and computational approaches require extensive screening of catalytic materials, often involving high economic and time costs. ML-based methods can help overcome these limitations by efficiently analyzing large datasets, identifying key performance trends, and predicting promising catalyst candidates with reduced experimental effort. Among ML approaches, unsupervised learning techniques such as clustering are particularly valuable, as they provide a structured way to analyze heterogeneous catalyst datasets.^342^ Given the complexity of electrochemical NRR, where the interplay of CC, FE, and PR, already highlighted in the previous sections, creates nontrivial trade-offs, clustering offers a holistic means of comparison that complements conventional analysis. To address the challenge of inconsistent reporting across studies, production rates were normalized within each experimental methodology and converted into a point-based system, enabling relative ranking of catalysts on a unified scale. Building on this framework, clustering was applied to identify groups of catalysts with comparable performance, based on normalized PR, FE, and CC. It should be emphasized that this approach constitutes a qualitative, exploratory analysis—designed to reveal relative trends and recurring patterns, rather than to provide quantitatively comparable benchmarks across all methods.^343,344^

#### 4.3.1 Clustering technique

*k*-Means clustering is one of the most widely used algorithms in unsupervised machine learning due to its simplicity and efficiency.^345^ Its core principle is to partition a dataset into *k* clusters by minimizing the distance between data points and their respective cluster centroids. The algorithm iteratively updates the position of each centroid until the optimal grouping of data points is achieved. In this way, catalysts with similar performance profiles can be grouped together, enabling a structured comparison across heterogeneous datasets. A critical step in applying *k*-means is the selection of the number of clusters (*k*). To evaluate clustering quality and determine the most suitable cluster count, the silhouette coefficient is frequently employed. These metric balances two aspects: (i) the average intra-cluster distance *a*(*i*), which measures how closely an object is grouped with others in its cluster, and (ii) the nearest inter-cluster distance *b*(*i*), which measures how far the object is from members of the closest neighboring cluster. The silhouette coefficient for an object *i* is defined as:4.5
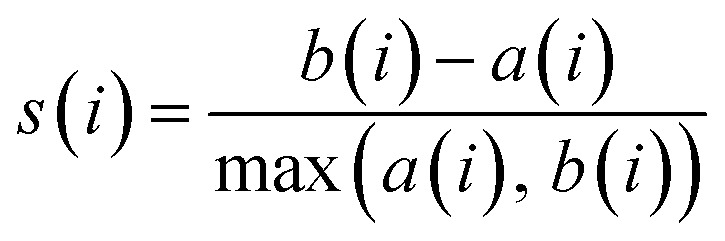


**Fig. 20:**
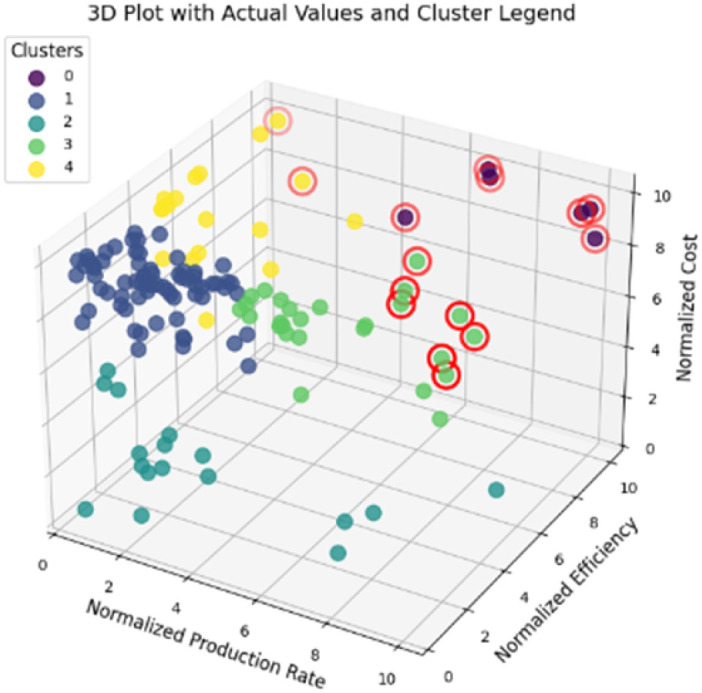
Clustering results obtained by applying *k*-means method to the catalyst data collection.

**Fig. 21:**
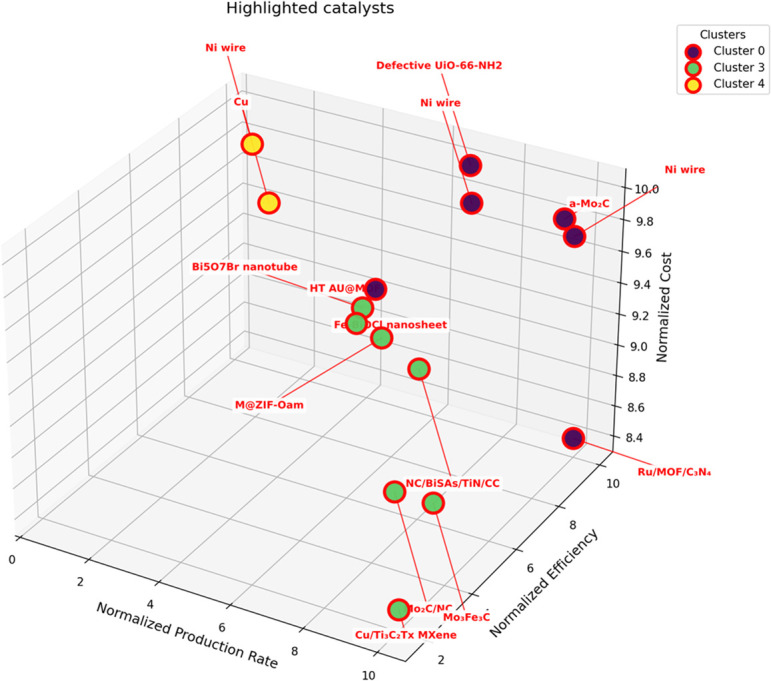
Detailed view of the relevant range of catalysts identified through the *k*-means method.

The value of *s*(*i*) ranges from −1 to 1. If *s*(*i*) approaches 1, the object is well-clustered and clearly separated from other groups. Values near 0 indicate overlapping clusters or ambiguous assignments, while negative values suggest misclassification. For visual interpretation, silhouette plots can be constructed (Fig. S1, SI), showing the distribution of *s*(*i*) across all clusters. Wide and clearly separated silhouettes indicate robust clustering, whereas narrow silhouettes reveal weak separation. By comparing silhouette coefficients across different values of *k*, the optimal cluster number can be identified. In addition, elbow plots (Fig. S2, SI) were generated to visually identify the point of diminishing returns in explained variance as *k* increases. In the context of eNRR catalyst analysis, clustering supported by silhouette evaluation provides a practical and interpretable means of detecting patterns, such as the recurrent grouping of certain metals or catalyst families in high-performing clusters. This approach complements conventional method-specific analysis and highlights trends that may guide future catalyst design efforts.^342,346^ Following the establishment of the clustering methodology, analysis was applied to the catalyst dataset to quantitatively elucidate underlying performance trends. The resulting 3D scatter plot (Fig. 20) depicts the clustering of the evaluated eNRR catalysts based on normalized PR, FE, and CC. Catalytic systems highlighted and labeled in red correspond to those achieving a total score exceeding 20 points, thereby defining the top-performing catalysts within the dataset (Fig. 21). Crucially, this normalization step solved one of the key challenges in catalyst comparison: the inconsistent reporting of performance metrics across studies. By converting absolute values into relative, unit-independent scores, heterogeneous datasets could be merged into a unified framework, enabling meaningful clustering and ensuring that observed trends truly reflect intrinsic catalyst performance rather than methodological bias. Importantly, the scoring procedure is fully unit-independent, as all input parameters were normalized prior to clustering. This normalization eliminates potential bias arising from differences in the absolute magnitudes of production rates, efficiencies, or costs, ensuring a consistent and objective ranking of catalysts.

The top-scoring catalysts identified through this data-driven approach closely match those previously highlighted in the qualitative analysis, independently confirming their superior performance. For direct comparison, these catalysts are summarized in Table 9, including their calculated scores, key performance metrics, and qualitative classification. This overview provides a clear framework for assessing the correspondence between quantitative and qualitative evaluations and for prioritizing candidates for further experimental validation. Notably, the strong correspondence between the ML-based clustering results and the qualitative selection confirms that this unit-independent scoring approach can reliably highlight promising candidates, suggesting that similar ML-assisted strategies may accelerate catalyst screening in future studies.

**Table 9:** Overview of catalytic systems achieving the highest performance scores among the evaluated datasets

Entry	Catalyst	Method	Score (points)
16	Ni wire	LimNRR	29.4
22	Cu	LimNRR	20.1
30	Mo_2_C/NC	Electrocatalysis	21.0
32	NC/BiSAs/TiN/CC	Electrocatalysis	22.6
34	Defective UiO-66NH_2_	Electrocatalysis	26.9
35	HT-Au@MOF	Electrocatalysis	22.7
58	Mo_3_Fe_3_C	Electrocatalysis	22.3
60	M@ZIF-OAM	Electrocatalysis	22.2
86	a-Mo_2_C	Electrocatalysis	29.1
109	Cu/Ti_3_CT_*x*_ Mxene	Electrocatalysis	20.5
116	Fe-BiOCl-nanosheet	Photocatalysis	21.1
117	Bi_5_O_7_Br-nanotube	Photocatalysis	21.4
132	Ru/MOF/C_3_N_4_	Photocatalysis	28.4

While machine-learning approaches hold significant potential for accelerating the discovery of catalytic systems, their effectiveness critically depends on the availability of high-quality, standardized data. To this end, we propose a minimal set of reporting requirements for NRR publications, including: (i) ammonia production rates reported in a unified metric (µmol cm^−2^ s^−1^) to enable direct cross-catalyst comparison, (ii) faradaic efficiency, (iii) turnover frequency (TOF), (iv) electrochemically active surface area (ECSA), and, where available, complementary physical surface area metrics (*e.g.*, BET) (v) stability metrics expressed in hours of continuous or cycling operation, and, where feasible, (vi) catalyst cost. Collecting these data systematically as SI would allow the establishment of an open-access database, enabling machine-learning algorithms to directly exploit the dataset. Such a resource could accelerate the rational design and discovery of catalysts not only for NRR, but also for related reactions such as HER and CO_2_RR, bridging the gap between experimental reporting and computational screening.

### 4.4 Rational catalyst design guidelines

**Fig. 22:**
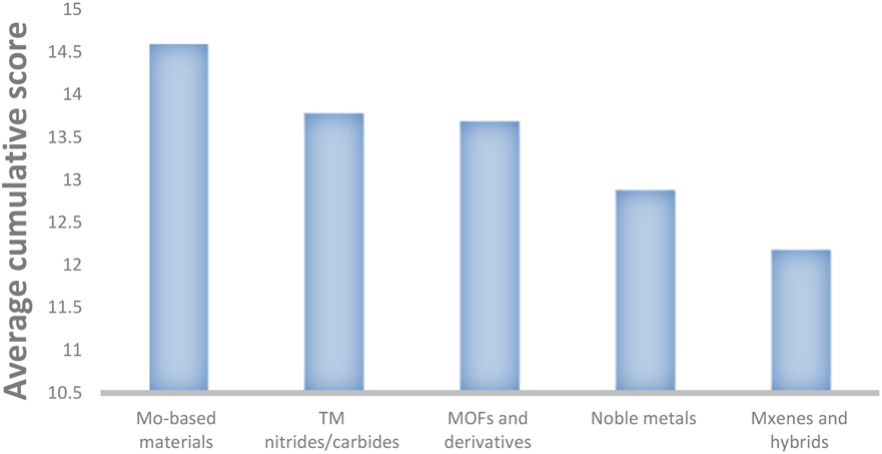
Average cumulative scores of representative catalyst classes for electro-chemical NRR.

Based on the combined quantitative evaluation and ML-assisted analysis, this section distills the key insights into practical design guidelines for NRR catalysts. To provide a concise overview, 140 representative catalytic systems were grouped into five major material classes: Mo-based catalysts, MOF-based hybrids, MXenes and their composites, transition-metal nitrides/carbides, and noble-metal-based materials. To provide an insightful overview of the evaluated catalytic systems, Fig. 22 summarizes the average cumulative performance scores of the different catalyst classes, highlighting recurring features associated with high activity and favorable cost–performance. Mo-based catalysts, transition-metal nitrides/carbides, as well as MOF-based systems and their derivatives consistently populate the top-performing range, reflecting their balanced activity, elemental abundance, and tunable coordination environments. These characteristics render them particularly suitable as foundational platforms for electrocatalytic NRR. In contrast, MXenes and MXene-based hybrids, as well as noble-metal-based catalysts, generally exhibit less favorable overall performance, predominantly due to their limited abundance and higher material costs. This class-based comparison confirms that future catalyst development must carefully balance catalytic performance, faradaic efficiency, and material cost to enable realistic scale-up and practical implementation.

## 5 Conclusion and outlook

This review provides a comprehensive overview of the current state-of-the-art methods for NRR, with particular emphasis on the electrochemical pathway. Special attention is devoted to catalytic materials and systems, since catalysis remains a crucial factor for enabling efficient scale-up toward practical NH_3_ production. To systematically explore trends in catalyst design, a dataset of 215 catalytic systems was compiled and analyzed. A key insight from this effort was the inconsistency of reported data, particularly the frequent omission of values such as TOF or stability, and the lack of uniformity in the units used for production rates. To address these issues, a normalization procedure was applied, enabling unit-independent comparison across catalytic systems. The resulting dataset was subsequently evaluated through a combined approach of qualitative/quantitative manual assessment and quantitative analysis employing machine learning techniques. These analyses enabled the identification of key trends in catalyst design, highlighting the critical role of transition-metal centers (*e.g.*, Mo, Ni, Cu), conductive high-surface-area supports, including materials such as MOFs and MXenes, and structural tailoring through porosity, defect engineering, and heteroatom doping. Such characteristics consistently correlate with enhanced performance, providing a clear framework to guide the rational development of catalysts for both fundamental research and industrial applications. In line with this framework, Mo-based catalysts, transition-metal nitrides and carbides, as well as MOFs and their derivatives emerge as particularly promising material classes, as they combine high intrinsic activity with material abundance, structural tunability, and favorable cost–performance characteristics. These systems therefore represent a strong basis for future electrocatalyst development in electrochemical NRR. To accelerate catalyst development for the NRR, improvements in the standardization of data reporting are essential. It is emphasized that a fundamental, consistent unit should be adopted to represent catalytic activity in terms of ammonia production. We propose using a molecular unit per time and catalyst surface area, such as µmol cm^−2^ s^−1^. In addition, TOF should be included as an intrinsic descriptor, as it reflects the activity per active site and provides complementary information on catalyst performance. Furthermore, we propose systematic reporting of key parameters, particularly when evaluating catalysts or catalytic systems. In this context, the electrochemically active surface area (ECSA), complemented by physical surface area measurements (BET) when available, along with TOF and stability expressed in hours, are essential descriptors for adequately characterizing a catalytic system. The absence of such standardized and comprehensive data not only hampers direct comparison between catalysts but also limits the application of advanced computational approaches. Machine learning, in particular, has the potential to significantly accelerate catalyst screening and identify key trends in design parameters. Its effectiveness, however, depends on the availability of high-quality, well-curated datasets. As highlighted by this review, current data fragmentation can severely restrict the generalization of the obtained conclusions. Nevertheless, the potential of ML methods is enormous, and, with consistent and standardized reporting, they fully realize their capabilities, enabling the targeted development and optimization of NRR catalysts.

## Conflicts of interest

The authors declare no conflict of interest.

## Abbreviations

ADP: Adenosine diphosphate
AEL: Alkaline electrolysis
AEM: Anion exchange membrane
ATP: Adenosine triphosphate
BET: Brunauer–Emmett–Teller
CB: Conduction band
CC: Catalyst cost
CCS: Carbon capture and storage
Cp_2_TiCl_2_: Titanocen-dichlorid
DFT: Density functional theory
DOE: Department of energy
*E*_a_
: Activation energy
ECSA: Electrochemically active surface area
EtOH: Ethanol
FE: Faradaic efficiency
GJ: Gigajoule
HB: Haber–Bosch
HER: Hydrogen evolution reaction
ILs: Ionic liquids
kWh: Kilowatt-hour
LiBF_4_: Lithiumtetrafluoroborat
Li-mNRR: Lithium-mediated nitrogen reduction reaction
LiPF_6_: Lithiumhexafluorophosphat
LiTFSI: Lithiumbis(trifluormethylsulfonyl)imid
LH_2_: Liquefied hydrogen
LMeOH: Liquefied methanol
LNH_3_: Liquefied ammonia
MOC: Metal–organic compound
MOF: Metal–organic framework
MPa: Megapascal
NRR: Nitrogen reduction reaction
NTP: Non-thermal plasma
P/Pi: Phosphate/inorganic phosphate
PEC: Photoelectrocatalysis
PEM: Proton exchange membrane
PEMEL: Proton exchange membrane electrolyzer
PR: Production rate
PSA: Pressure swing adsorption
PV: Photovoltaic
QDs: Quantum dots
SEM: Scanning electron microscopy
SMR: Steam methane reforming
SOE: Solid oxide electrolysis
SSC: Stainless steel cloth
TCPP: Tetrakis(4-carboxyphenyl)porphyrin
THF: Tetrahydrofuran
t: metric ton
TOF: Turnover frequency
TRL: Technology readiness level
VB: Valence band
Δ*G*: Free energy difference

## Supplementary Material

NA-OLF-D5NA01170A-s001

## Data Availability

All data analysed in this review are taken from previously published studies and are available in the cited literature. The compiled dataset used for comparison is provided in the manuscript and in the supplementary information (SI). Supplementary information is available. See DOI: https://doi.org/10.1039/d5na01170a.
