# Supplementary material for: Catalyst screening for electrochemical ammonia synthesis: a critical review
Source: Nanoscale Adv. 2026 Apr 27;8(10):3035–72. doi: 10.1039/d5na01170a (PMC13112091; doi:10.1039/d5na01170a)
Supplement: NA-008-D5NA01170A-s001 [file NA-008-D5NA01170A-s001.pdf]

Supplementary Information (SI) for Nanoscale Advances.
This journal is © The Royal Society of Chemistry 2026

Supplementary Information for

Catalyst Screening for Electrochemical Ammonia Synthesis: A Review

Dominik G. Jammal[*1], **Ricardo Bernardino**[1], **Nuno Canha**[1] **Cristina M. Cordas**[1, 2], **Rui P.P.L. Ribeiro**[1, 2]

[1] HyLab- Green Hydrogen Collaborative Laboratory Sines Central Termoelétrica, Estrada Nacional 120-1, 7520-089, Sines, Portugal
[2] LAQV-Requimte, Department of Chemistry, NOVA School of Science and Technology, NOVA University of Lisbon, Caparica, Portugal

* Correspondence: dominik.jammal@hylab.pt

Supporting Information – Selection of Catalytic Systems

The 215 catalytic systems analyzed in this review were systematically compiled from recent review articles to ensure comprehensive and up-to-date coverage of Lithium-mediated, electrochemical, and photo(electro)catalytic nitrogen reduction reactions. The following review publications served as the primary sources for the initial compilation of catalytic systems.[1 – 7] Each system extracted from these reviews was cross-checked against the original primary literature referenced in the respective tables to verify reported performance metrics, including NH_3 production rates, Faradaic efficiencies, and, where available, catalyst costs. Only systems with consistent and verifiable data were included to ensure reproducibility and reliability.

Table S 1: *Comprehensive Overview of 215 Catalytic Systems for N_2 Reduction: Production Rates (PR), Faradaic Efficiencies (FE), Catalyst Costs (CC), and combined Performance Scores.*

	Catalysts	PR (normalized)	FE (normalized)	Costs	Sum of Points	Review Source
1	**Mo foil**	0.53	1.68	5.13	7.3	1
2	**Mo rod**	n/a	1.45	8.44	9.9	1
3	**Cu foil**	0.8	2.71	9.83	13.3	1
4	**steel cloth**	2	4.15	9.89	16.0	1
5	**Au-coated carbon paper**	0.57	4.15	0.5	5.2	1
6	**SSC**	n/a	6.49	9.89	16.4	1
7	**Steel cloth**	0.5	4.56	9.89	15.0	1
8	**Ag wire**	0.56	1.75	8.09	10.4	1
9	**Cu wire**	0.51	6.04	9.85	16.4	1
10	**Mo foil**	0.57	4.33	9.66	14.6	1

11	**Mo foil**	0.56	4.33	9.66	14.6	1
12	**Ni wire**	0.62	9.1	9.77	19.5	1
13	**Ni wire**	0.61	10	9.77	20.4	1
14	**Mo foil**		3.52	9.66	13.2	2
15	**Ni wire**	7	9.91	9.77	26.7	2
16	**Ni wire**	10	9.64	9.77	29.4	2
17	**Cu(HBT)**	1.5	7.57	7.23	16.3	2
18	**Mo foil**	n/a	6.04	9.66	15.7	2
19	**Mo foil**	n/a	8.11	9.66	17.8	2
20	**Mo foil**	0.58	4.6	9.66	14.8	2
21	**Cu (HBT)**	0.55	2.35	7.23	10.1	2
22	**Cu**	3	7.21	9.85	20.1	2
23	**Mo foil**		8.02	9.66	17.7	2
24	**Cu wire**	0.7	4.78	9.85	15.3	2
25	**Fe**	0.52	6.22	9.88	16.6	2
26	**Hollow Cr_2O_3 microspheres**	3.55	1.16	9.84	14.55	3
27	**TiO_2-GO**	2.31	0.76	9.98	13.05	3
28	**Fe_2O_3 nanorods**	2.41	0.5	9.95	12.86	3
29	**Defect-rich MoS_2 nanoflower**	4.03	1.33	9.98	15.34	4
30	**Mo_2C/NC**	10	1.78	9.2	20.98	4
31	**V_2CTx MXene**	2	0.88	2.55	5.43	4
32	**NC/BiSAs/TiN/CC**	9.73	3.17	9.74	22.64	4
33	**JUC-1000**	10	1.74	7	18.74	5
34	**Defective UiO-66-NH2**	6.89	10	9.99	26.88	5
35	**HT AU@MOF**	5.98	7.26	9.48	22.72	5
36	**MoS2@ZIF-71**	1.92	3.88	8.76	14.56	5
37	**$Bi_2V_0.1O_x/CeO_2$**	3.29	1.54	9.98	14.81	3
38	**B_4C**	3.7	2.19	9.99	15.88	3
39	**Nb_2O_5 nanofiber**	5.78	1.44	9.95	17.17	3
40	**Cr_2O_3/CPE**	3.9	1.36	9.9	15.16	4
41	**TA-reduced Au/TiO_2**	3.08	1.31	7.57	11.96	4
42	**α-Au/CeO_2-RGO**	1.49	1.53	7.13	10.15	3
43	**Au flowers**	3.58	1.08	9.58	14.24	3
44	**Mo_2C@3DUM-C**	4.18	1.46	9.8	15.44	4
45	**Au/Ti_3C_2**	4.19	2.46	7.41	14.06	4
46	**1T-MoS_2/Ti_3C_2**	4.9	0.99	9.57	15.46	4
47	**Cr_3C_2@CNFs**	3.38	1.36	9.99	14.73	4
48	**Ti_3C_2Tx (T_{14}F,OH) MXene nanosheets**	2.96	1.44	7.52	11.92	4

49	**Fluorine-free Ti_3C_2Tx ($T_{14}O$,OH)**	4.96	1.42	9.8	16.18	4
50	**TiO_2/Ti_3C_2Tx**	3.67	1.34	2.5	7.51	4
51	**Hydroxyl-rich Ti_3C_2Tx QDs**	8.11	1.89	2.55	12.55	4
52	**Oxygen-vacancy-rich TiO_2/Ti_3C_2Tx**	4.37	2.21	6.45	13.03	4
53	**Fe-TCPP**	3.91	2.22	9.38	15.51	5
54	**OPA-PCN-222(Fe)**	1.61	2.33	6.94	10.88	5
55	**Mo_2N Nanorods**	10	0.9	8.6	19.5	4
56	**β-FeOOH nanorods**	3.31	1.15	9.99	14.45	3
57	**Mo_2C/C**	1.85	1.27	9.95	13.07	4
58	**Mo_3Fe_3C**	10	3.44	8.9	22.34	4
59	**Co3HHTTP2**	3.16	0.77	9.5	13.43	5
60	**M@ZIF-Oam**	7.37	5.35	9.5	22.22	5
61	**a-FeB2 PNSs**	5.32	2.28	8.29	15.89	
62	**Ni nanoparticles/V_4C_3Tx MXene**	3.06	2.07	2.55	7.68	4
63	**W_2N_3**	1.89	1.71	9.47	13.07	4
64	**Ti_3C_2 MXene nanoribbons**	2.27	0.74	2.5	5.51	4
65	**Cu@Ce-MOF-2**	5.92	1.61	9.57	17.1	5
66	**Co3Fe-MOF**	6.5	3.28	0.5	10.28	5
67	**Au nanorods**	0.67	0.83	9.93	11.43	3
68	**γ-Fe_2O_3**	0.5	0.61	10	11.11	3
69	**NeS-doped Ti_3C_2Tx**	4.64	1.14	8.11	13.89	4
70	**N-doped porous carbon**	3.37	0.55	8.92	12.84	3
71	**CNT@CAU-17**	2.09	3.92	6.21	12.22	5
72	**Co-TCPP**	2.86	1.69	9.78	14.33	5
73	**Zn-TCPP**	6.52	1.11	6.95	14.58	5
74	**NiFe-MOF**	1.55	1.69	6.95	10.19	5
75	**NCNT@CAU-17**	0.93	2.64	9.66	13.23	5
76	**CNT@UIO-66**	1.22	2.1	9.66	12.98	5
77	**NCNT@UIO-66**	0.97	2.44	8.24	11.65	5
78	**CNT@BIT-58**	1.47	1.79	8.24	11.5	5
79	**NCNT@BIT-58**	1.14	2.08	1.84	5.06	5
80	**NCNT@MIL-101(Fe)**	1.32	3.23	1.84	6.39	5
81	**CNT@MIL-101(Fe)**	3.6	4.6	8.29	16.49	5
82	**TiO_2/Ti**	0.979	0.98	9.92	11.879	3
83	**MoS_2/CC**	0.92	0.69	9.82	11.43	3
84	**MnO/TM**	1.08	2.18	10	13.26	4
85	**Fe_3O_4/Ti**	0.5	1	9.91	11.41	3

86	**a-Mo_2C**	10	9.12	9.94	29.06	4
87	**TiN-PE**	2.24	2.4	9.61	14.25	4
88	**ZIF-67@Ti3C2**	10	4.8	2.13	16.93	5
89	**In-MOF**	1.86	3.69	8.6	14.15	5
90	**H-KUST**	0.89	0.97	5.25	7.11	5
91	**MoO_3 nanosheet**	3.01	0.85	9.52	13.38	3
92	**Mo nanofilm**	0.7	0.59	9.88	11.17	3
93	**Ru/C**	0.51	0.5	9.52	10.53	3
94	**VN/TM**	0.93	0.92	8.91	10.76	3
95	**Ag nanosheet**	0.74	1.48	9.86	12.08	3
96	**MoN**	0.51	0.68	7.64	8.83	3
97	**MoN NA/CC**	2.07	0.68	0.76	3.51	4
98	**TiB2**	1.41	2.91	9.88	14.2	4
99	**VN/CC**	1.79	1.21	6.75	9.75	4
100	**MV-MoN@NC**	7.08	1.93	0.5	9.51	4
101	**Mo_2N**	2.91	1.41	9.85	14.17	4
102	**PEBCD/C**	0.63	1.05	9.6	11.28	3
103	**AuHNCs**	0.83	0.5	8.3	9.63	3
104	**Surface-engineered Ti_3C_2**	0.64	1.96	4.24	6.84	4
105	**NH2–MIL-88B–Fe**	1.13	3.14	0.5	4.77	3
106	**Au nanorod**	0.51	1.3	9	10.81	3
107	**Ru/Ti**	1.12		8.7	9.82	3
108	**VN**	2.23	1.74	8.93	12.9	4
109	**Cu/Ti_3C_2Tx MXene**	10	2.02	8.43	20.45	4
110	**Ag-Au@ZIF**	0.55	4.35	7.75	12.65	5
111	**CrO0.66N0.56**	0.96	1.93	7.5	10.39	4
112	**Au@ZIF-8**	2.96	10	6.6	19.56	5
113	**Fe_2O_3–CNT**	0.51	0.47	0.5	1.48	3
114	**CP_2TiCl_2**	5.48	0.48	9.86	15.82	3
115	**Fe-BiOBr nanosheet (1)**	6.36	0.44	9.99	16.79	6
116	**Fe-BiOCl nanosheet (2)**	8.4	2.67	9.99	21.06	6
117	**Bi5O7Br nanotube**	8.16	3.29	9.99	21.44	6
118	**Bi2MoO6 sphere**	8.17	1.34	9.98	19.49	6
119	**CuCr-LDH nanosheet**	2.41	0.98	9.98	13.37	6

120	**MoO3−x nanosheet**	6.11	0.82	9.51	16.44	6
121	**Ti3C2Tx/TiO2**	6.49	0.5	9.6	16.59	6
122	**WO3**	4.09	0.59	9.98	14.66	6
123	**P-C3N4**	1.6	1.68	10	13.28	6
124	**Au-$Ru_{0.31}$ Nanokristalle**	3.43	0.7	7.83	11.96	6
125	**Mo-$W_{18}O_{49}$ Ultrathin Nanowires**	5.16	0.85	9.94	15.95	6
126	**Au/TiO_2-OV**	4.54	1.46	9.72	15.72	6
127	**CuCr-LDH Nanosheets**	2.79	0.56	9.98	13.33	6
128	**SAFe-porous g-C_3N_4**	2.51	0.56	9.95	13.02	7
129	**Co-doped Bi_2MoO_6**	3.37	1.7	9.37	14.44	7
130	**Fe/Zr-MOFs**	2.27	1.13	9.39	12.79	7
131	**COFX Au**	6.66	0.8	9.33	16.79	6
132	**Ru/MOF/C_3N_4**	9.99	10	8.42	28.41	6
133	**PCN-V**	0.88	1.68	9.98	12.54	6
134	**IN2S3-X@ZnS**	2.53	0.57	9.63	12.73	6
135	**Ag-Pt/TiO2**	1.87	0.59	9.69	12.15	6
136	**POM(PMo10V2) and MOF(MIL-88-A)**	2.3	0.8	9.94	13.04	6
137	**CEF3/LiNbO3**	2.94	0.82	9.66	13.42	6
138	**Ru-KzTa2O6-x**	1.24	1.38	8.44	11.06	6
139	**C3N4/MoS2/Mn3O4 SVs**	5.03	1.93	9.71	16.67	6
140	**B-C3N4 (MoO2)**	8.2	1.21	9.97	19.38	6
141	**Au/TiO2**	2.96	1.46	9.72	14.14	6
142	**Au/g-C3N4 hollow sphere**	6.45	1.23	9.64	17.32	6
143	**Ru-CoS/g-C3N4 SVs**	6.53	2.02	9.59	18.14	6
144	**BiOBr nanosheet (1)**	2.44	n/a	9.99	12.43	6
145	**Bi5O7Br nanostructure**	10	n/a	9.99	19.99	6
146	**Bi2MoO6/BiOBr**	3.3	n/a	9.97	13.27	6
147	**H-Bi5O7I**	5.5	n/a	9.98	15.48	6
148	**$Cu^{δ+}$-ZnAl-LDH nanosheet**	3.46	n/a	9.98	13.44	6
149	**FeS2-FeP-CeO2**	9.65	n/a	9.96	19.61	6
150	**In2O3/In2S3 microsphere**	2.08	n/a	9.53	11.61	6
151	**GaN (Ru) NVs**	6.52	n/a	8.5	15.02	6
152	**Ultrathin MoS2 SVs**	6.06	n/a	9.97	16.03	6
153	**FeN-CDs/TiO_2@CN**	6.35	n/a	9.99	16.34	7

154	**Al-PMOF(Fe)**	0.5	n/a	9.98	10.48	7
155	**Pt_1/N-MoS_2**	3.88	n/a	3.26	7.14	7
156	**Fe-BiOCl Nanosheets**	8.4	n/a	9.96	18.36	7
157	**Au/$(BiO)_2CO_3$**	2.03	n/a	9.64	11.67	7
158	**5%Ru@n-GaN NWs**	3.86	n/a	8.3	12.16	7
159	**Cs_2O/Os-Au**	9.61	n/a	0.5	10.11	7
160	**UiO-66(-NH_2)/$CuInS_2$**	7.08	n/a	6	13.08	7
161	**$FeIN_2S_4$/Fe-Pal**	7.56	n/a	9.55	17.11	7
162	**Bi_2S_3/OV-Bi_2MoO_6**	3.91	n/a	9.96	13.87	7
163	**Cu-Cu_2O/CMOH**	5.47	n/a	9.78	15.25	7
164	**Co-doped Bi_2MoO_6 (1)**	4.53	n/a	9.37	13.9	7
165	**Cu-doped Bi_2MoO_6 (2)**	5.63	n/a	9.37	15	7
166	**Bi–MOF/g-C_3N_4**	5.45	n/a	9.99	15.44	7
167	**Cu_2O Clusters/MIL-100(Fe)**	2.33	n/a	9.93	12.26	7
168	**BiOBr/OV-TiO_2-Cu**	5.05	n/a	9.96	15.01	7
169	**S-doped-g-C_3N_4**	9.93	n/a	10	19.93	7
170	**Carbon-WO_3·H_2O**	5.56	n/a	9.98	15.54	7
171	**ZnO/ZnSnO3/Carbon Dots**	9.74	n/a	9.99	19.73	7
172	**TiO2/BiOBr**	8.11	n/a	9.99	18.1	7
173	**Bi2SN2O7/BiOBr**	6.59	n/a	9.98	16.57	7
174	**Boron-doped graphene quantum dots/Bi2MoO6**	5.48	n/a	9.36	14.84	7
175	**2D/2D Bi12O17Br2/ZnCr-LDH**	6.2	n/a	0.85	7.05	7
176	**N-graphyne/Bi/BiOBr**	1.61	n/a	9.87	11.48	7
177	**p-TiO2**	2.63	n/a	10	12.63	7
178	**Bi2S3@PCN**	1.08	n/a	9.98	11.06	7
179	**NanoMIL-125(Ti)**	5.46	n/a	10	15.46	7
180	**COF/g-C3N4/CNT**	5.21	n/a	9.51	14.72	7
181	**NiSnO3-g-C3N4**	7.47	n/a	9.93	17.4	7
182	**Sb/TiO2**	1.33	n/a	9.99	11.32	6
183	**Few-layer g-C3N4 NVs**	9.97	n/a	10	19.97	6
184	**NC-g-C3N4**	8.22	n/a	10	18.22	6
185	**MOF-74(Zn)@DF-C3N4**	8.35	n/a	9.99	18.34	6
186	**S-g-C3N4 nanosheet CVs**	9.93	n/a	9.99	19.92	6

187	**WS2@TiO2 film**	8.18	n/a	9.92	18.1	6
188	**B-g-C3N4 Nanosheet**	6.35	n/a	9.98	16.33	6
189	**B-g-C3N4**	6.5	n/a	9.98	16.48	6
190	**YF3+/ATP nanocomposite**	2.12	n/a	9.65	11.77	6
191	**In(OH)3/g-C3N4**	9.58	n/a	9.65	19.23	6
192	**Fe-SrMoO4**	3.4	n/a	9.93	13.33	6
193	**Cyano group/g-C3N4**	9.49	n/a	9.99	19.48	6
194	**In2S3 nanotube SVs**	2.36	n/a	9.48	11.84	6
195	**Pr3+: CeF3/ATP (attapulgite)**	7.55	n/a	9.82	17.37	6
196	**AuRuNPs**	3.43	n/a	6.96	10.39	6
197	**Bi2O2CO3 nanosheet**	4.67	n/a	9.96	14.63	6
198	**TiO2 nanotubular**	7.29	n/a	9.98	17.27	6
199	**O-g-C3N4 NVs**	9.88	n/a	10	19.88	6
200	**S-g-C3N4 NVs**	3.14	n/a	10	13.14	6
201	**1T-MoS2/CdS SVs**	10	n/a	8.74	18.74	6
202	**Mo0.1Ni0.1Cd0.8S SVs**	2.72	n/a	7.79	10.51	6
203	**Zn0.1Sn0.1Cd0.8S SVs**	3	n/a	6.4	9.4	6
204	**Holey g-C3N4 nanosheet CVs**	6.41	n/a	9.74	16.15	6
205	**g-C3N4/ZnMoCdS SVs**	2.87	n/a	8.47	11.34	6
206	**Sn-doped MOF-5**	5.13	n/a	9.81	14.94	7
207	**TiO2 QDs/ Fe3S4**	9.36	n/a	9.93	19.29	7
208	**g-C3N4/rGO**	4.57	n/a	7.38	11.95	7
209	**Bi2O3/CdMoO4**	3.33	n/a	8.11	11.44	7
210	**g-C3N4/MoS2/PbTiO3**	9.72	n/a	8.97	18.69	7
211	**NaNbO3/Bi2O2CO3**	4.13	n/a	7.85	11.98	7
212	**TiO2 QDsmodified Bi2O3/NaBiS2**	7.12	n/a	9.77	16.89	7
213	**TCN/ZnS/ZnIN2S4**	7.04	n/a	0.5	7.54	7
214	**Ag-δ-Bi_2O_3**	0.5	n/a	7.69	8.19	7
215	**g-C3N4/Ag2CO3 heterojunction NVs**	4.77	n/a	8	12.77	7

S2. Machine Learning Analysis

The Supplementary Information includes the Silhouette plots, Elbow plots, and a parallel coordinates plot that were used to guide the machine learning clustering procedure. The Silhouette and Elbow plots were applied to quantitatively evaluate cluster cohesion and separation, enabling the identification of the optimal number of clusters. The parallel coordinates plot provides a complementary visualization of the

multidimensional relationships among the catalytic systems and their performance metrics. Together, these analyses document the methodological steps leading to the final clustering solution and support a clear and transparent interpretation of the machine learning outcomes.

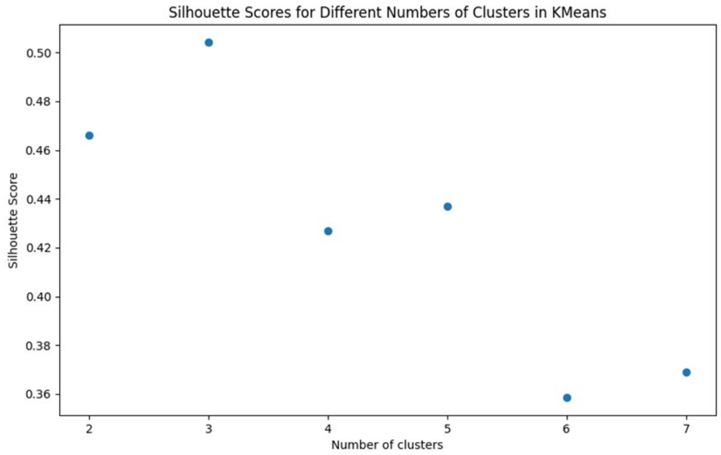

Figure S1: *Silhouette Scores for different numbers of Clusters*

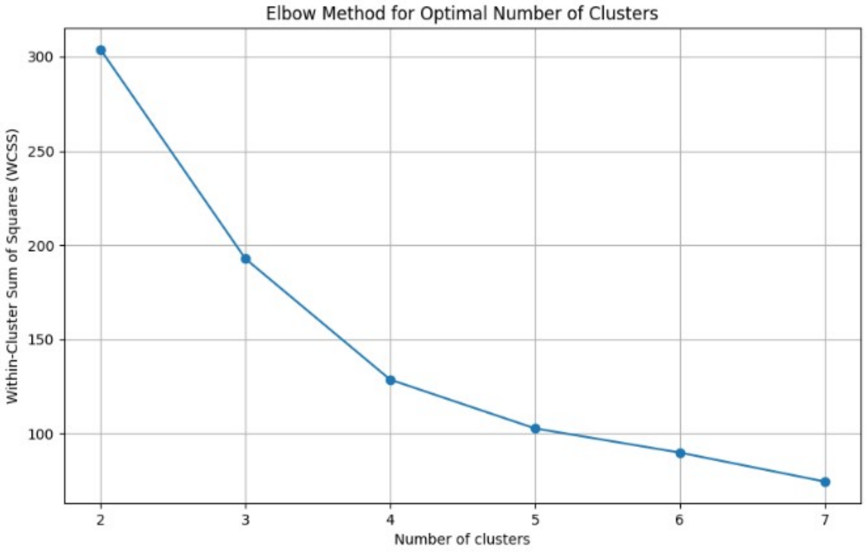

Figure S2: *Elbow Method for Determining the Optimal Number of Clusters*

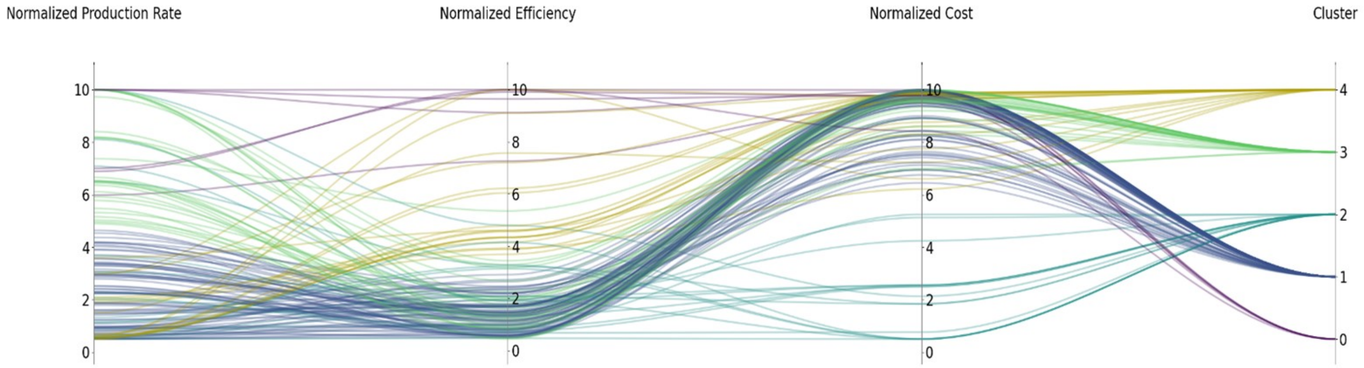

Figure S3: *Visualization of Catalytic Systems Using Parallel Coordinates Plot.*

References

1. Ahmed MI, Assafiri A, Hibbert DB, Zhao C. Li-Mediated Electrochemical Nitrogen Fixation: Key Advances and Future Perspectives. *Small*. 2023;19(52). doi:10.1002/smll.202305616

2. Mangini A, Fagiolari L, Sacchetti A, Garbujo A, Biasi P, Bella F. Lithium-Mediated Nitrogen Reduction for Ammonia Synthesis: Reviewing the Gap between Continuous Electrolytic Cells and Stepwise Processes through Galvanic Li–N_2 Cells. *Adv Energy Mater*. 2024;14(25). doi:10.1002/aenm.202400076

3. Mahmood S, Iqbal S, Wang Z, et al. Emerging electrocatalysts for green ammonia production: Recent progress and future outlook. *Arabian Journal of Chemistry*. *Elsevier B.V.* 2024;17(10). doi:10.1016/j.arabjc.2024.105950

4. Biswas A, Bhardwaj S, Boruah T, Dey RS. Electrochemical ammonia synthesis: fundamental practices and recent developments in transition metal boride, carbide and nitride-class of catalysts. *Mater Adv*. 2022;3(13):5207-5233. doi:10.1039/D2MA00279E

5. He H, Wen HM, Li HK, Zhang HW. Recent advances in metal–organic frameworks and their derivatives for electrocatalytic nitrogen reduction to ammonia. *Coord Chem Rev*. 2022;471:214761. doi:10.1016/j.ccr.2022.214761

6. Shen H, Yang M, Hao L, Wang J, Strunk J, Sun Z. Photocatalytic nitrogen reduction to ammonia: Insights into the role of defect engineering in photocatalysts. *Nano Res*. 2022;15(4):2773-2809. doi:10.1007/s12274-021-3725-0

7. Ješić D, Pomeroy B, Kamal KM, Kovačič Ž, Huš M, Likozar B. Photo- and Photoelectrocatalysis in Nitrogen Reduction Reactions to Ammonia: Interfaces, Mechanisms, and Modeling Simulations. *Advanced Energy and Sustainability Research*. Published online June 16, 2024. doi:10.1002/aesr.202400083
